# Dual roles of influenza B virus neuraminidase mRNA vaccine in enhancing cross-lineage protection by supplementing inactivated split vaccination

**DOI:** 10.1128/jvi.02294-24

**Published:** 2025-04-23

**Authors:** Chau Thuy Tien Le, Ki-Hye Kim, Jannatul Ruhan Raha, Noopur Bhatnagar, Surya Sekhar Pal, Phillip Grovenstein, Mahmuda Yeasmin, Rong Liu, Bao-Zhong Wang, Sang-Moo Kang

**Affiliations:** 1 Center for Inflammation, Immunity and Infection, Institute for Biomedical Sciences, Georgia State University 439338, Atlanta, Georgia, USA; St. Jude Children's Research Hospital, Memphis, Tennessee, USA

**Keywords:** NA mRNA-LNP, split IBV vaccines, cross-protection, influenza virus

## Abstract

The current influenza vaccine is based on immunity to hemagglutinin (HA) and provides poor cross-protection. Here, we generated mRNA vaccine encoding influenza B virus (IBV) neuraminidase (NA) conjugated to influenza A virus M2 ectodomain (M2e), encapsulated in lipid nanoparticles (LNP), capable of inducing cross-lineage IBV protection in a dose-dependent pattern. The combination of low-dose NA mRNA and inactivated split IBV vaccines was found to induce significantly higher levels of cross-reactive IgG responses, NA and HA inhibition titers, effector and memory cellular immune responses as well as cross-lineage protection than either NA mRNA or split vaccine alone. This study suggests that the NA mRNA vaccine not only provides cross-lineage protection with a high dose but also enhances the cross-protective efficacy of the combined low-dose NA mRNA and split vaccines. Our findings support a new strategy of using mRNA LNP-supplemented conventional vaccination to enhance cross-protection.

**IMPORTANCE:**

This study highlights a significant advancement in influenza vaccination strategies. To test a new vaccination strategy, we developed an influenza B virus (IBV) neuraminidase (NA) mRNA vaccine which could provide cross-lineage protection at a high dose. More importantly, the co-administration of NA mRNA and split IBV vaccine at low doses was found to significantly enhance the hemagglutinin and NA immunity as well as cross-lineage protection of seasonal IBV vaccines. This proof-of-concept study provides evidence for a novel strategy to enhance the immunogenicity and cross-protective efficacy of conventional vaccines by supplementing with new targets of mRNA vaccines.

## INTRODUCTION

The most common seasonal influenza vaccine is an inactivated split virion, inducing strain-specific neutralizing immunity against hemagglutinin (HA) with a highly variable nature. The overall seasonal vaccine effectiveness is low (10%–60%) because of HA receptor binding site mutations in circulating strains (1, 2). The second major influenza surface protein neuraminidase (NA) has been recognized as a potential target for inducing cross-protective immunity, independent of HA-head antibodies (3, 4). Influenza A virus M2 ion channel protein ectodomain (M2e) provides a broadly cross-protective antigen with relatively weak efficacy (5–7). Broad protection against influenza A and B viruses was demonstrated with virus-like particle (VLP) vaccine displaying multi-subtype consensus NA and 5xM2e repeat proteins in mice (8) and ferrets (9). Despite broad protection by influenza universal vaccine candidates targeting NA and M2e, the effectiveness has been limited due to non-neutralizing immunity. Also, the VLP vaccines might have issues with scaling up production, purification, and stability (10).

The success of COVID-19 vaccines based on mRNA has gained much attention in developing new mRNA vaccine strategies. Immunization of mice with H1 HA mRNA (10–90 µg mRNA intramuscular route, 3–30 µg mRNA intradermal route) induced homo protection with dose-dependent variable weight loss (11). Substantial weight loss against heterologous virus challenge was reported in mice even with a high dose (10–50 µg) and multi-subtype HA mRNA vaccines (11, 12). Combination of multi-antigen targeting mRNA vaccines (5 µg of each NA, HA-Stalk, M2, and NP mRNA) or pentavalent (including HA) mRNA vaccines could provide enhanced homo and heterologous protection (13–15). A vehicle of lipid nanoparticles (LNP) to deliver mRNA vaccines was reported to be inflammatory when used with high doses (LNP to encapsulate 10 µg mRNA) (16, 17). This inflammatory nature of empty LNP (an equivalent dose for 30 µg mRNA) was shown to have adjuvant effects on recombinant HA protein (10 µg) co-vaccination (11). It remains unknown whether a low dose of mRNA LNP vaccine would exhibit immune-enhancing effects.

In this study, we designed a new cross-protective mRNA construct encoding influenza B virus (IBV) NA conjugated to 4xM2e tandem repeat (NA mRNA) as a proof-of-concept test. This study showed that a high dose (3 µg) of IBV NA mRNA vaccine was required to be immunogenic and capable of broad IBV protection. In addition, we hypothesized that the combination of low-dose NA mRNA and inactivated split vaccines would enhance immune responses and cross-protective efficacy, compared to either low-dose mRNA or split vaccine alone. Here we report a notable finding that the combination of low-dose NA mRNA (1 µg) and split vaccines enhanced humoral and cellular immune responses as well as cross-lineage IBV protection. Overall, our findings support a new strategy of immunization with the combination of mRNA and conventional vaccines at low doses to enhance the immunogenicity and cross-protective efficacy.

## RESULTS

### Construction of influenza B virus chimeric NA mRNA vaccine

**Fig 1:**
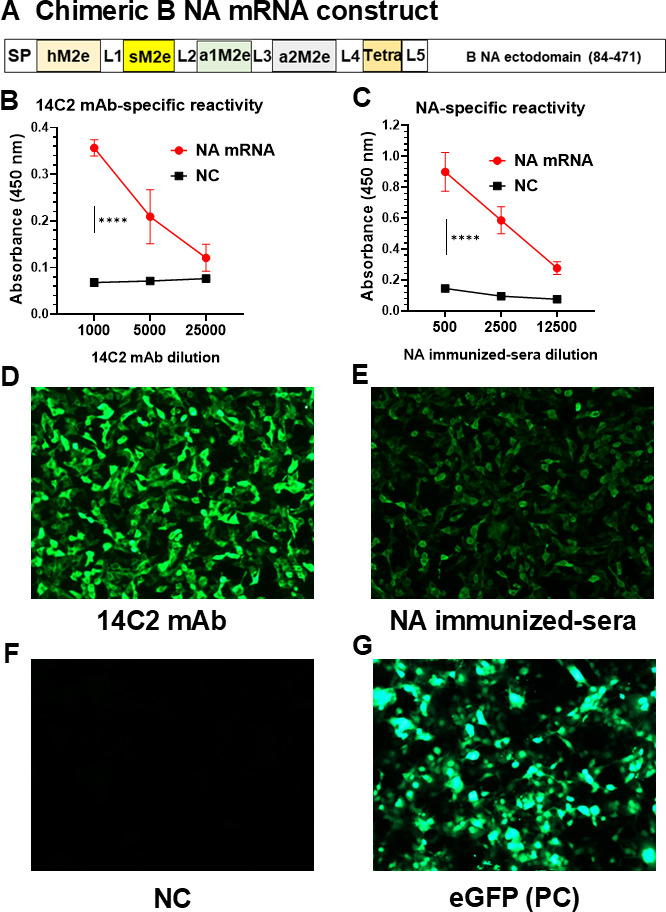
Influenza B virus chimeric NA mRNA vaccine expresses M2e-NA conjugate protein. (**A**) Chimeric B NA mRNA construct design. Each domain amino acid (aa) sequence information is provided in Fig. S1. (**B–G**) HEK 293T cells were transfected with chimeric NA mRNA or eGFP mRNA to characterize protein expression. The reactivity to M2e and NA expressed by B NA mRNA vaccine construct was measured using 14C2 monoclonal antibody (mAb) (**B**) and NA-specific polyclonal antisera (**C**) by ELISA. NC: negative (no mRNA) control. (**D, E**) Expression of chimeric NA protein by NA mRNA transfected HEK 293T cells was assessed under a fluorescence microscope, with no mRNA transfection as negative control (**F**), eGFP mRNA transfection as positive control (PC) (**G**). All results are presented as mean ± standard error mean (SEM) with individual dots. Statistical analysis was performed using two-way ANOVA and Tukey’s post-multiple comparison tests. *P*-value is significant at *P* < 0.05; ****P < 0.0001.

We constructed IBV NA mRNA vaccine encoding a chimeric NA composed of signal peptide tPA, 4xM2e (hM2e–sM2e–a1M2e–a2M2e), and tetramer stabilizing domain conjugated to consensus NA full-length amino acids 84–471 ectodomain (named NA mRNA for simplicity, Fig. 1A; Fig. S1). The chimeric B NA mRNA sequence was codon-optimized by increasing G/C contents and minimizing uridine nucleosides. NA mRNA vaccine was prepared through *in vitro* transcription where uridines were replaced with N1-methylpseudouridine. The expression of B NA mRNA was confirmed in HEK 293T cells transfected with B NA mRNA by cell surface enzyme-linked immunosorbent assay (ELISA) using M2e and NA specific antibody reactivity (Fig. 1B and C) and fluorescent microscope (Fig. 1D through G).

### Influenza B virus chimeric NA mRNA (3 µg dose) vaccination induces cross-reactive IgG antibodies and provides broad cross-protection

**Fig 2:**
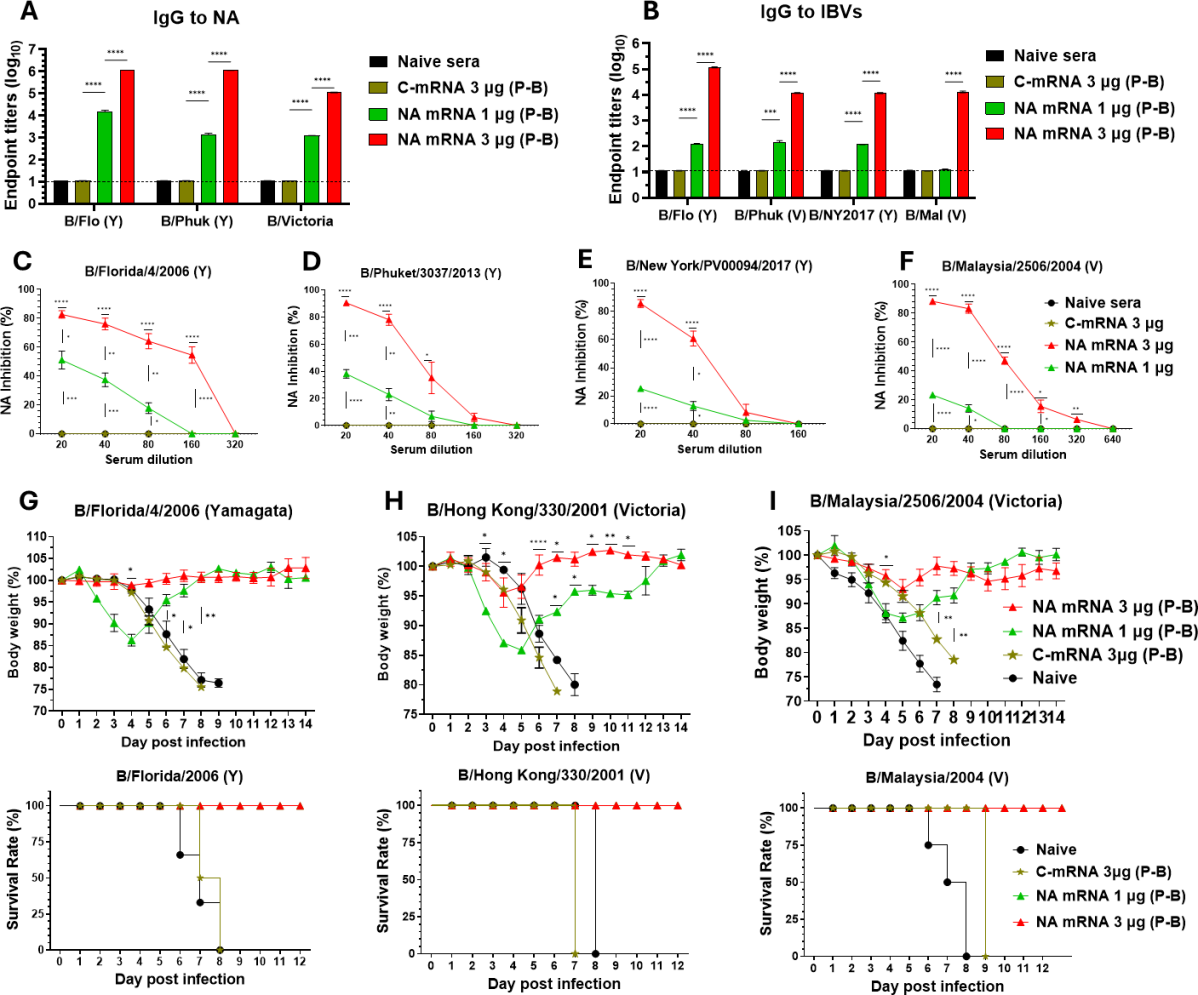
NA mRNA LNP vaccine confers cross-lineage protection against IBV in a dose-dependent pattern. Mice were vaccinated (i.m., *n* = 10) with NA mRNA (1 or 3 µg) LNP or Control (SARS-CoV-2 spike) mRNA (C-mRNA 3 µg) at two doses (prime-boost, 4 weeks interval). (**A, B**) IgG antibody responses (P-B: prime-boost sera) to NA proteins from different IBV strains (**A**) and inactivated IBV antigens (**B**). (**C–F**) NAI (*n* = 5) activities against B/Florida/4/2006 (Y), B/Phuket/3037/2013 (Y), B/New York City/PV00094/2017 (Y), and B/Malaysia/2056/2004 (V) in boost sera. (**G–I**) Weight changes and survival rates in vaccinated and control mice after challenge with B/Florida/2006 (Y, 1.07 × 10^5^ EID_50_, *n* = 3), B/Hong Kong/330/2001 (V, 1.4 × 10^5^ EID_50_, *n* = 3), B/Malaysia/2004 (V, 1.58 × 10^5^ EID_50,_ *n* = 5). All results are presented as mean ± standard error mean (SEM) with individual dots. Statistical analysis was performed using two-way ANOVA and Tukey’s post-multiple comparison tests. *P*-value is significant at *P* < 0.05; *P < 0.0332, **P < 0.0021, ***P < 0.0002, ****P < 0.0001.

Chimeric B NA mRNA was encapsulated into LNP, which was used in all mice for vaccination to investigate the immunogenicity and efficacy of NA mRNA LNP vaccine *in vivo*. After the prime (first) dose (3 µg) of NA mRNA vaccination, low levels of IgG antibodies specific for NA of IBV (B/Florida) were induced (Fig. S2). NA mRNA (3 µg) boost vaccination generated significantly increased levels of NA-specific IgG titers (~10^6^ for NA of B/Florida and B/Phuket, ~10^5^ for NA of B/Victoria/705/2018; Fig. 2A) and IgG titers against viral antigens (Fig. 2B) derived from Yamagata (Y) (~10^5^ for B/Flo, ~10^4^ for B/NY) and Victoria (V) (~10^4^ for B/Phuket and B/Malaysia) lineage strains, which were higher by 2–3 log10 folds than those in the NA mRNA 1 µg group. Control (SARS-CoV-2 spike) mRNA (C-mRNA 3 µg) vaccination did not induce IgG antibodies specific for influenza antigens. IgG antibodies specific for M2e (~10^5^ IgG titers for hM2e, sM2e, a1M2e, a2M2e) were induced at high levels after the boost dose of chimeric NA mRNA (3 µg) vaccination (Fig. S3A). NA mRNA (3 µg) boosted antisera exhibited higher levels of NAI (NA inhibition) titers (~160 IC_50_) against B/Flo (Y) (Fig. 2C), moderate NAI titers (~80 IC_50_) against B/Phuket (Y) (Fig. 2D) and B/Malaysia (V) and a low but significant level of NAI (<80 IC_50_) against B/NY (Y) (Fig. 2E and F). Low-dose NA mRNA (1 µg) vaccination induced low or negligible levels of NAI activity titers compared to the high dose (3 µg) NA mRNA vaccine group (Fig. 2C through F).

Furthermore, to determine B NA mRNA protective efficacy, we challenged the vaccinated and control mice with different lineage IBVs at lethal doses and measured body weight (BW) changes and survival rates. Boost vaccination with NA mRNA (3 µg) induced protection against B/Florida/4/2006 (Y, 0% BW loss), B/Hong Kong/330/2001 (V, ~5% BW loss), and B/Malaysia/2004 (V, ~7% BW loss) as evidenced by preventing severe weight loss and achieving 100% survival rates (Fig. 2G through I). In contrast, the NA mRNA 1 µg group provided survival protection with substantial weight loss against B/Florida/4/2006 (15% BW loss), B/Hong Kong/330/2001 (~15% BW loss), and B/Malaysia/2004 (V, ~13% BW loss). The C-mRNA group was not protected against IBV challenge and did not survive, similar to the naïve mice after infection (Fig. 2G through I).

Since the chimeric B NA mRNA vaccine construct is conjugated to 4xM2e (Fig. 1A) and thus raised IgG antibodies specific for M2e, we performed *in vivo* protection tests of antisera against influenza A virus (Fig. S3B) as described (18, 19). The naïve mice that were intranasally inoculated with a mixture of antisera and A/Nanchang/1995 H3N2 were protected against weight loss, whereas the control naïve mice inoculated with unvaccinated sera and A/Nanchang/1995 virus displayed severe weight loss and died of infection (Fig. S3B). Collectively, chimeric NA mRNA vaccination effectively induced cross-reactive IgG antibodies for NA and viral antigens, functional NAI titers, and protection against both Y and V lineage IBVs in a dose-dependent pattern.

### The combination of low-dose (1 µg) mRNA-LNP and split vaccines significantly enhances cross-lineage binding IgG titers

**Fig 3:**
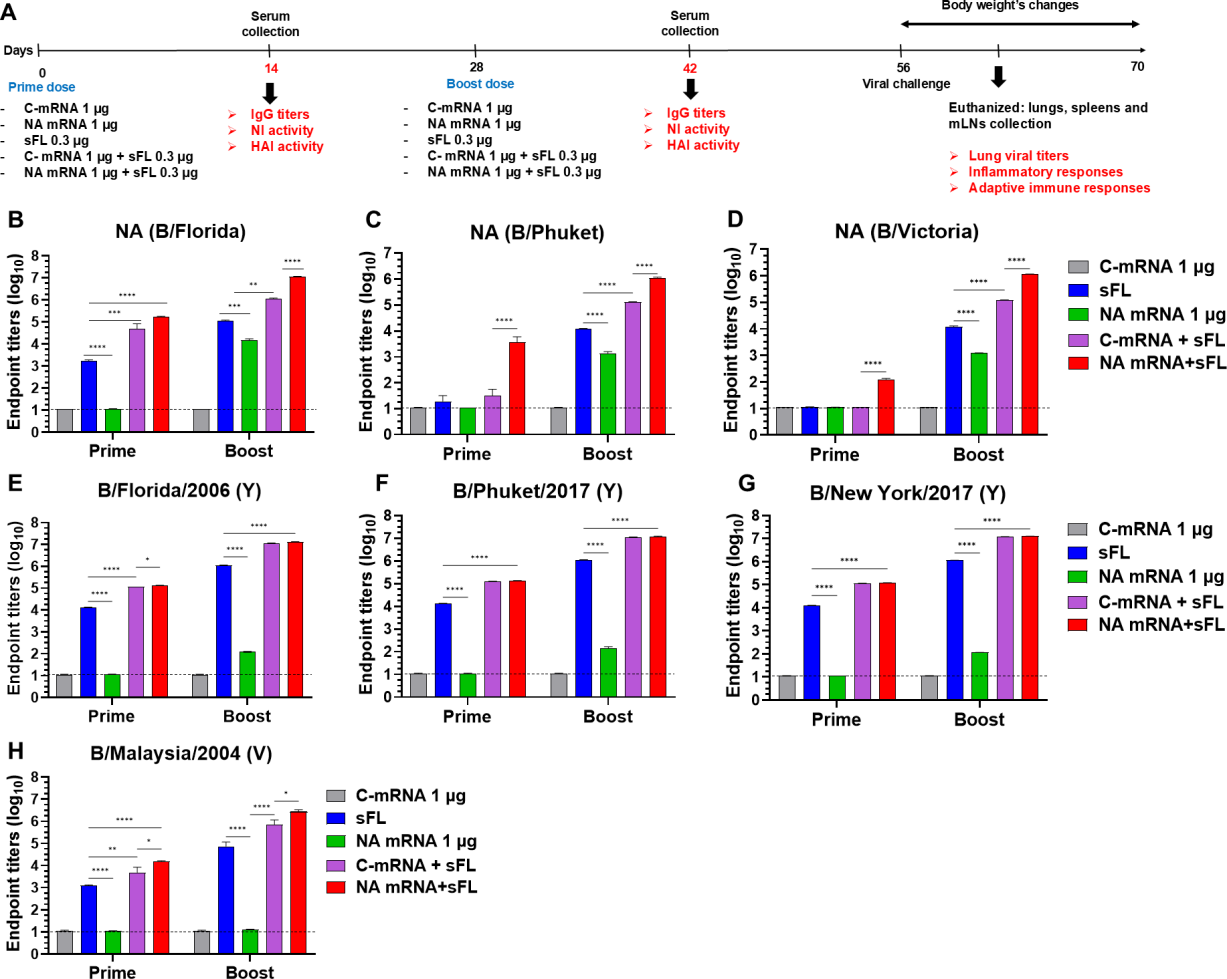
Low-dose NA mRNA + sFL vaccination enhances IgG to NA and IBV. (**A**) Vaccine groups, timeline for immunization and assays: Mice (*n* = 10) were vaccinated intramuscularly (i.m.) with sFL 0.3 µg ± 1 µg of NA mRNA LNP, NA mRNA 1 µg, or control (SARS-CoV-2 spike) mRNA 1 µg (C-mRNA) LNP. IgG antibodies to NA protein derived from different IBV strains post-prime and boost (**B–D**), inactivated IBVs (**E–H**) in boost sera. Statistical analysis was performed using two-way ANOVA and Tukey’s post-multiple comparison tests. *P-*value is significant at *P* < 0.05; *P < 0.0332, **P < 0.0021, ***P < 0.0002, ****P < 0.0001.

Next, we investigated whether the combination of low-dose NA mRNA-LNP and IBV split vaccines would enhance humoral immune responses in comparison with NA mRNA and split alone vaccines. Groups of mice were intramuscularly (i.m.) immunized with inactivated IBV split (sFL 0.3 µg), B NA mRNA (1 µg), C-mRNA (1 µg), combination split (sFL 0.3 µg) plus B NA mRNA (1 µg) or sFL 0.3 µg plus C-mRNA (1 µg) as described in Fig. 3A. The combination groups (NA mRNA + sFL, C-mRNA +sFL) significantly enhanced the levels of IgG titers (10^5^, ~ 7 × 10^4^ for NA of B/Flo) compared to the sFL alone and NA mRNA alone groups after a prime dose. The sFL vaccine was more effective in inducing IgG titers (~10^3^) to NA of B/Flo than B-NA mRNA (Fig. 3B). It is notable that the NA mRNA + sFL group (but not the C-mRNA +sFL group) showed substantial levels of IgG for NA from B/Phuket (Y) and B/Victoria/705/2018 (5 × 10^3^ and 10^2^ titers, respectively) post prime (Fig. 3C and D). Consistently, the NA mRNA + sFL group raised the highest levels of 10^7^ and 10^6^ IgG titers against NA of B/Florida, B/Phuket, and B/Victoria, respectively, followed by the groups of C-mRNA + sFL (10^6^ to 10^5^ titers), sFL (10^5^ to 10^4^ titers), and B NA mRNA (10^4^ to 10^3^ titers) after boost (Fig. 3B through D). Overall, the NA mRNA + sFL group exhibited approximately 10-fold higher levels of IgG reactive to NA proteins derived from hetero- and cross-lineage strains than the C-mRNA + sFL group, suggesting the contribution of NA mRNA (1 µg) to enhancing NA-specific IgG levels.

When IgG levels for IBV antigens were determined, the combination groups (NA mRNA + sFL, C-mRNA + sFL) showed 10-fold higher levels of IgG titers (10^5^) for B/Florida (Y), B/Phuket (Y), B/NY/17 (Y), and IgG titers of 3 × 10^4^ for B/Malaysia (V) than those in the sFL alone group after prime and then increased approximately 100-fold in IgG titers after boost vaccination (Fig. 3E through H). It is also noted that the NA mRNA + sFL group induced a moderately higher level of IgG to cross-lineage B/Malaysia (V) than the C-mRNA + sFL (Fig. 3H). B-NA mRNA vaccination at a low dose (1 µg) was not effective in inducing IgG titers against IBV antigens (Fig. 3E through H). The NA mRNA + sFL combination group (but not the NA mRNA only group) induced a moderate level of IgG (1–8 × 10^3^ titers) specific for hM2e, a1M2e, a2M2e, and sM2e after boost (Fig. S4A).

### The combination of low-dose (1 µg) mRNA-LNP and split vaccines significantly enhances functional cross-reactive neuraminidase and hemagglutination inhibition activities

**Fig 4:**
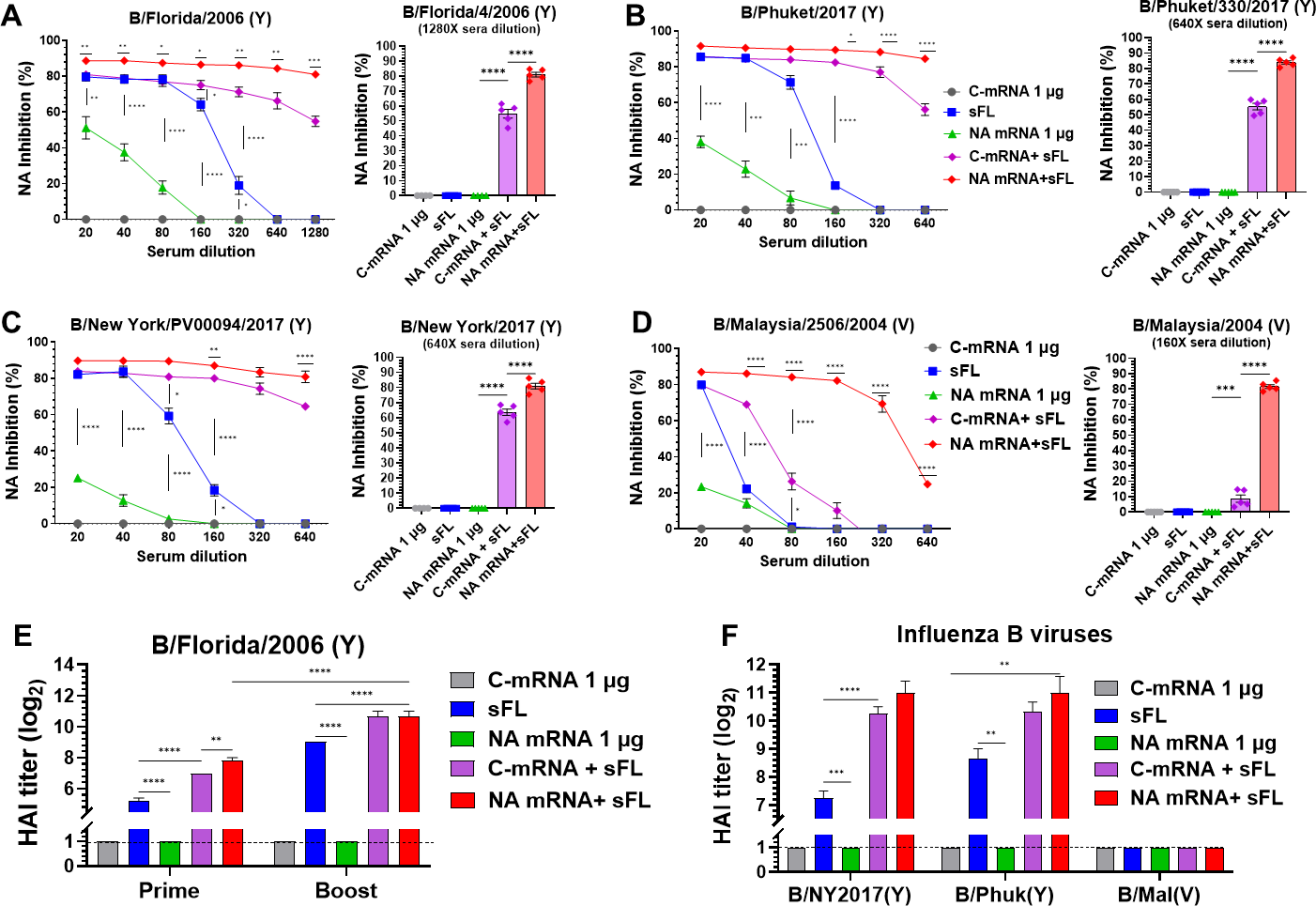
Low-dose NA mRNA + sFL vaccination enhances cross-lineage NAI and homo- and hetero HAI titers. The vaccine groups are the same as in Fig. 3. (**A–D**) NAI titers in serial serum dilutions and NAI comparisons by bar graphs at a representative dilution. (**A**) NAI against B/Florida/2006 (Y) at serial and 1280× dilutions. (**B**) NAI against B/Phuket/2013 (Y) at serial and 640× dilutions. (**C**) NAI against B/New York/2017 (Y) at serial and 640× dilutions. (**D**) NAI against B/Malaysia/2004 (V) at serial and 160× dilutions. (**E**) HAI titers against B/Florida/2006 (Y) in prime and boost sera. (**F**) HAI titers against B/New York/2017 (Y), B/Phuket/2013 (Y), and B/Malaysia (V) in boost sera. All results (*n* = 5 mice/group) are presented as mean ± standard error mean (SEM) with individual dots. Statistical analysis was performed using one-way ANOVA and Tukey’s post-multiple comparison tests (**A–D**), two-way ANOVA and Tukey’s post-multiple comparison tests (**A–F**). *P*-value is significant at *P* < 0.05; *P < 0.0332, **P < 0.0021, ***P < 0.0002, ****P < 0.0001.

Regarding the induction of functional antibodies, boost antisera of NA mRNA + sFL vaccination consistently showed the highest levels of NAI activity against B/Florida (Y), B/Phuket (Y), B/NY/17 (Y), and B/Malaysia (V), followed by the C-mRNA + sFL group (Fig. 4A through D). The NAI against B/Malaysia (V) was most prominent, resulting in 8 folds of difference between the NA mRNA + sFL and C-mRNA + sFL groups (Fig. 4D), suggesting the contribution of NA mRNA to enhancing NAI titers. The sFL (0.3 µg) alone and NA mRNA (1 µg) alone groups showed the lowest or negligible levels of NAI activity among the three vaccine groups (Fig. 4A through D).

Prime antisera of the NA mRNA + sFL group exhibited the highest level of HAI activity against B/Florida, which were 2- and 16-fold higher than those of C-mRNA + sFL and sFL alone prime sera, respectively (Fig. 4E). HAI activity against B/Florida was proportionally increased by approximately 8 folds after boost in three vaccine groups (Fig. 4E). Boost sera of the NA mRNA + sFL and C-mRNA + sFL groups showed approximately 8- and 4-fold higher levels of cross-HAI activities against homologous lineage B/New York City/2017 (Y) and B/Phuket (Y) viruses, respectively, than those of sFL vaccine (Fig. 4F). HAI activity against cross-lineage B/Malaysia (V) virus was not detected in antisera from any vaccine group (Fig. 4F).

To further test HA-specific antibody protective function *in vivo*, intranasal co-inoculation of anti-sera (prime-dose) and B/Florida/4/2006 virus to naïve mice was performed by monitoring weight changes and survival rates for 12 days (Fig. S5). Correlating with HAI titers observed in prime-dose sera, the naïve mice inoculated with a mixture of virus and NA mRNA + sFL or C-mRNA + sFL sera showed better protection against B/Florida virus (~3–5% weight loss) than sFL alone (~17% wt loss, 66% survival rate) and NA mRNA 1 µg alone prime dose (0% survival rate) vaccine-serum groups (Fig. S5).

Taken together, these results suggest that NA mRNA (1 µg) supplemented sFL vaccine more effectively enhanced the levels of NA- and M2e-specific IgG and homo- and hetero-reactive HAI activities within the same lineage strains and cross-lineage reactive NAI activity of humoral responses compared to C-mRNA + sFL vaccine. Both NA mRNA and C-mRNA, when co-immunized with sFL vaccine, displayed adjuvant effects on significantly enhancing homologous and heterologous IBV-specific IgG antibodies, cross-reactive NAI, and HAI activities.

### Combination NA mRNA plus split vaccine confers effective protection against homo and cross-lineage protection against IBV with clearing lung viral loads

**Fig 5:**
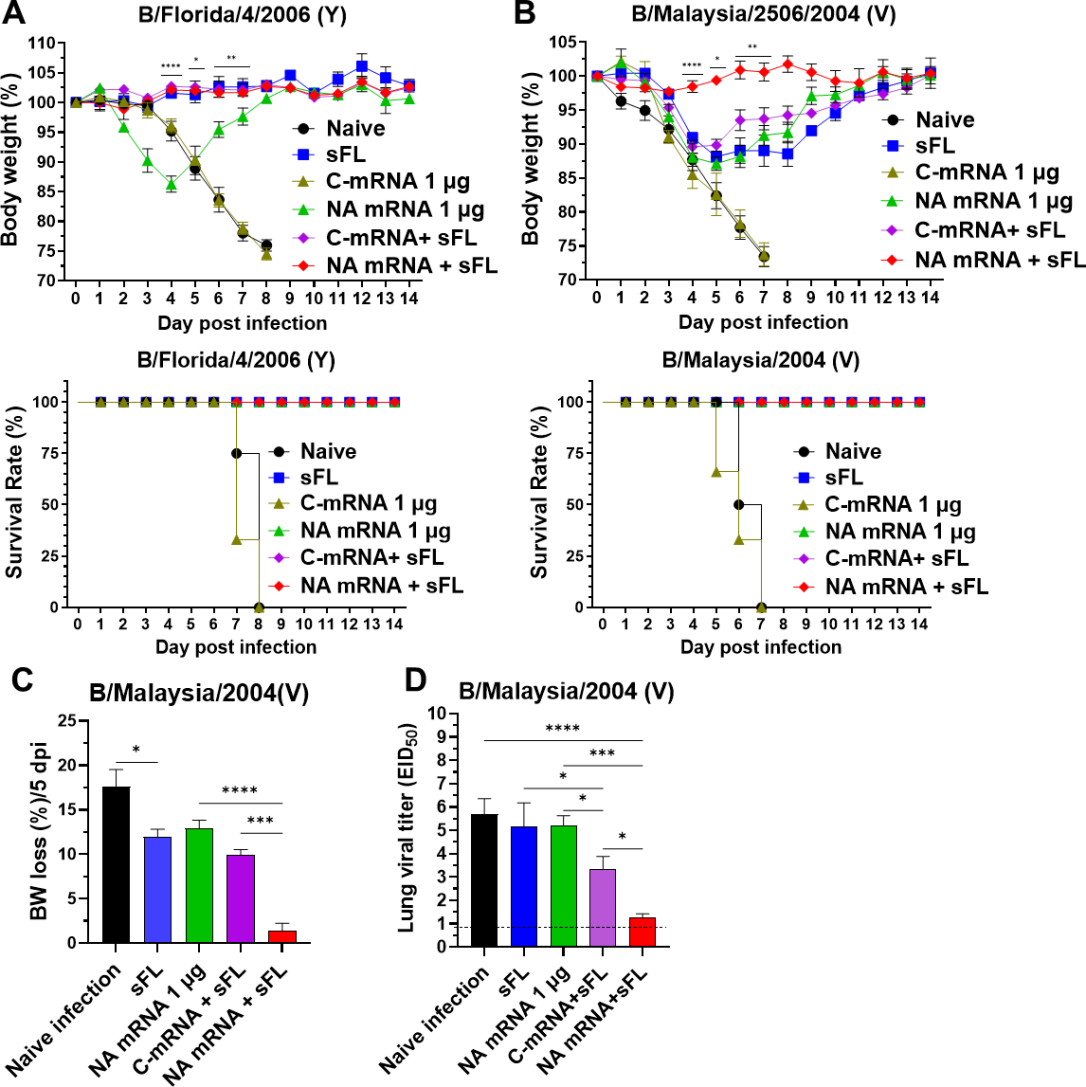
Low-dose NA mRNA + sFL co-vaccination induces enhanced cross-lineage protection. Immunization and experimental design were the same as in Fig. 3. (**A**) Body weight changes and survival rates in two-dose (boost)-vaccinated mice after intranasal (i.n.) challenge with homologous B/Florida/4/2006 virus (Y, 1.43 × 10^6^ EID_50_). (**B**) Weight changes and survival rates in boost-vaccinated mice following i.n. challenge with cross-lineage B/Malaysia/2506/2004 (V, 1.58 × 10^5^ EID_50_). (**C**) The percentages of BW loss in vaccinated and control mice reached a peak at 5 dpi with B/Malaysia/2004. (**D**) Lung viral titers in embryonated egg substrates at 5 dpi with B/Malaysia/2004. The data are presented as mean ± standard error mean (SEM) with individual dots. Statistical analysis was performed using one-way ANOVA and Tukey’s post-multiple comparison tests (**C, D**), two-way ANOVA and Tukey’s post-multiple comparison tests (**A, B**). *P*-value is significant at *P* < 0.05; *P < 0.0332, **P < 0.0021, ***P < 0.0002, ****P < 0.0001.

The sFL alone (0.3 µg), C-mRNA + sFL and NA mRNA + sFL boost dose groups were sufficient for conferring homologous protection in mice, whereas NA mRNA (1 µg) vaccinated mice displayed ~14% BW loss despite 100% survival rates (Fig. 5A). Mice with C-mRNA-LNP (mock control) died of infection after B/Florida/4/2006 virus infection (Fig. 5A). Antiserum of mice with prime (first) dose combination NA mRNA + sFL provided more effective protection against homologous B/Florida/4/2006 virus (Y) in naïve mice by preventing weight loss when passively infected with virus and antiserum than sFL alone and C-mRNA + sFL prime dose sera (Fig. S5), supporting the protective advantage by including NA mRNA in vaccination. C-mRNA (1 µg) immunization could not protect against IBV challenge, whereas the NA mRNA (1 µg) alone group provided survival protection (Fig. 5A and B). NA mRNA + sFL (Y) vaccination provided cross-lineage protection against B/Malaysia/2004 (V) virus, by preventing weight loss while the groups of combination C-mRNA + sFL, sFL alone, and NA mRNA alone showed 10%–13% weight loss on day 5 post-infection (5 dpi) with B/Malaysia/2004 (Fig. 5B and C). All these data support the contribution of B NA mRNA to protection against IBV.

At 5 dpi with B/Malaysia/2004, when weight loss reached a peak (Fig. 5C), lung viral loads were determined (Fig. 5D). The NA mRNA + sFL group cleared lung viral titers by 10^4^ and 10^2^ folds more effectively than the unvaccinated or sFL, NA mRNA 1 µg, and C-mRNA + sFL groups, respectively at 5 dpi (Fig. 5D). Also, the C-mRNA + sFL group showed ~90-fold lower viral titers than the sFL alone group, meanwhile NA mRNA group indicated similar viral titers as the sFL group.

### NA mRNA plus split sFL co-vaccination inducing effective protection results in suppressing post-infection lung inflammatory responses

**Fig 6:**
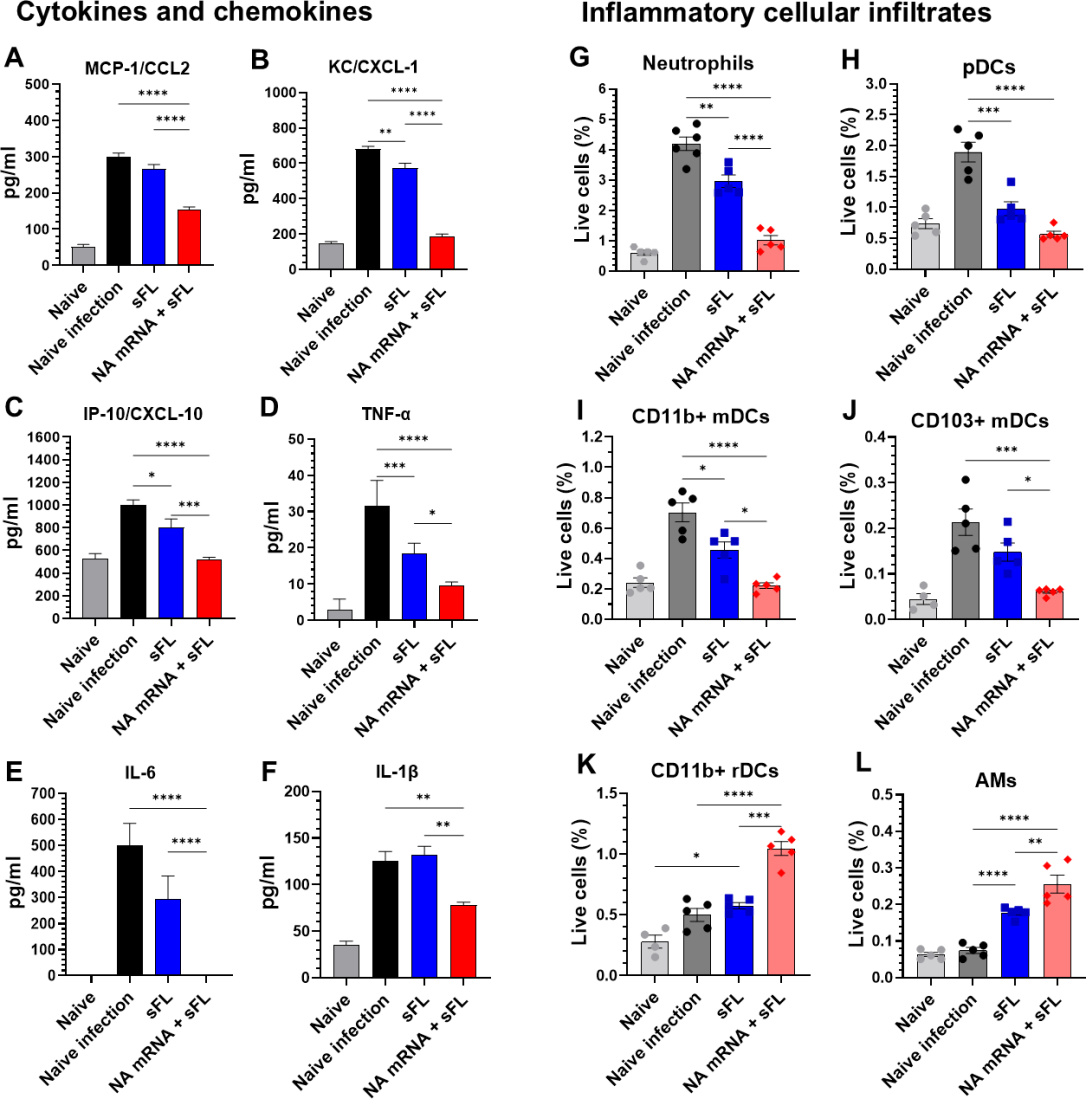
Low-dose NA mRNA + sFL vaccination protects from cross-lineage B/Malaysia virus-induced inflammatory responses. Vaccinated mice (i.m., *n* = 5) with NA mRNA 1 µg + sFL combination vaccines or sFL alone (Prime-Boost, 4 weeks interval) were challenged with B/Malaysia/2506/2004 (V) virus as described in Fig. 5. The lung samples were harvested on 5 dpi to determine inflammatory responses. (**A–F**) Inflammatory cytokines and chemokines in lung extracts at 5 dpi. (**G–L**) Inflammatory cellular infiltrates into the lungs. (**G**) Neutrophils: CD45^+^F4/80ˉCD11b^+^Ly6c^low^; (**H**) Plasmacytoid DCs (pDCs): CD45^+^F4/80ˉCD11c^+^MHC II^high^B220^+^; (**I**) CD11b^+^mDCs: CD45^+^F4/80ˉCD11c^+^MHC II^high^CD103⁻CD11b^+^; (**J**) CD103^+^mDCs: CD45^+^F4/80ˉCD11c^+^MHC II^high^CD103^+^CD11b⁻; (**K**) CD11b + resident DCs (rDCs): CD45^+^F4/80ˉCD11c^+^MHC II^low^ CD8a⁻CD11b^+^; (**L**) Alveolar macrophages (AMs): CD45^+^F4/80^+^CD11bˉCD11c^+^. All results are presented as mean ± standard error mean (SEM) with individual dots. Statistical analysis was performed using one-way ANOVA and Tukey’s post-multiple comparison tests. *P*-value is significant at *P* < 0.05; *P < 0.0332, **P < 0.0021, ***P < 0.0002, ****P < 0.0001.

Naïve mice after infection with B/Malaysia/2004 presented the highest levels of inflammatory chemokines, cytokines, and cellular infiltrates (Fig. 6). The NA mRNA + sFL group with clearance of viral loads showed the most effective suppression of monocyte chemoattractant protein-1 (MCP-1/CCL2), KC/CXCL-1, IP-10/CXCL-10, TNF-a, IL-6, and IL-1β in lung samples after challenge with B/Malaysia/2004 (Fig. 6A through F). After infection, all vaccine groups suppressed IFN-γ and IL-12p70 cytokines to lower levels than naïve-infected mice (Fig. S6A and B). Post-infection, naive mice displayed the highest lung infiltrations of neutrophils, plasmacytoid DCs (pDCs), CD11b^+^ migrating DCs (mDCs), CD103^+^ mDCs, eosinophils, monocytes, and monocyte-derived macrophages, whereas the NA mRNA + sFL group most effectively suppressed the infiltrations of these inflammatory cells into the lung close to a background (Fig. 6G through J; Fig. S6C through E) as determined by a multi-color flow gating strategy (Fig. S7). In contrast, lung resident CD11b^+^ rDCs and alveolar macrophages (AMs) were observed at higher levels in the NA mRNA + sFL group than those in the sFL and control groups (Fig. 6K and L). These results suggest that effective control of lung viral loads by NA mRNA + sFL vaccination protects from over-production of inflammatory cytokines and chemokines, and cellular infiltrates into the lung.

### NA mRNA plus sFL co-vaccination effectively induces B cell and T cell responses in lymphoid and lung tissues

**Fig 7:**
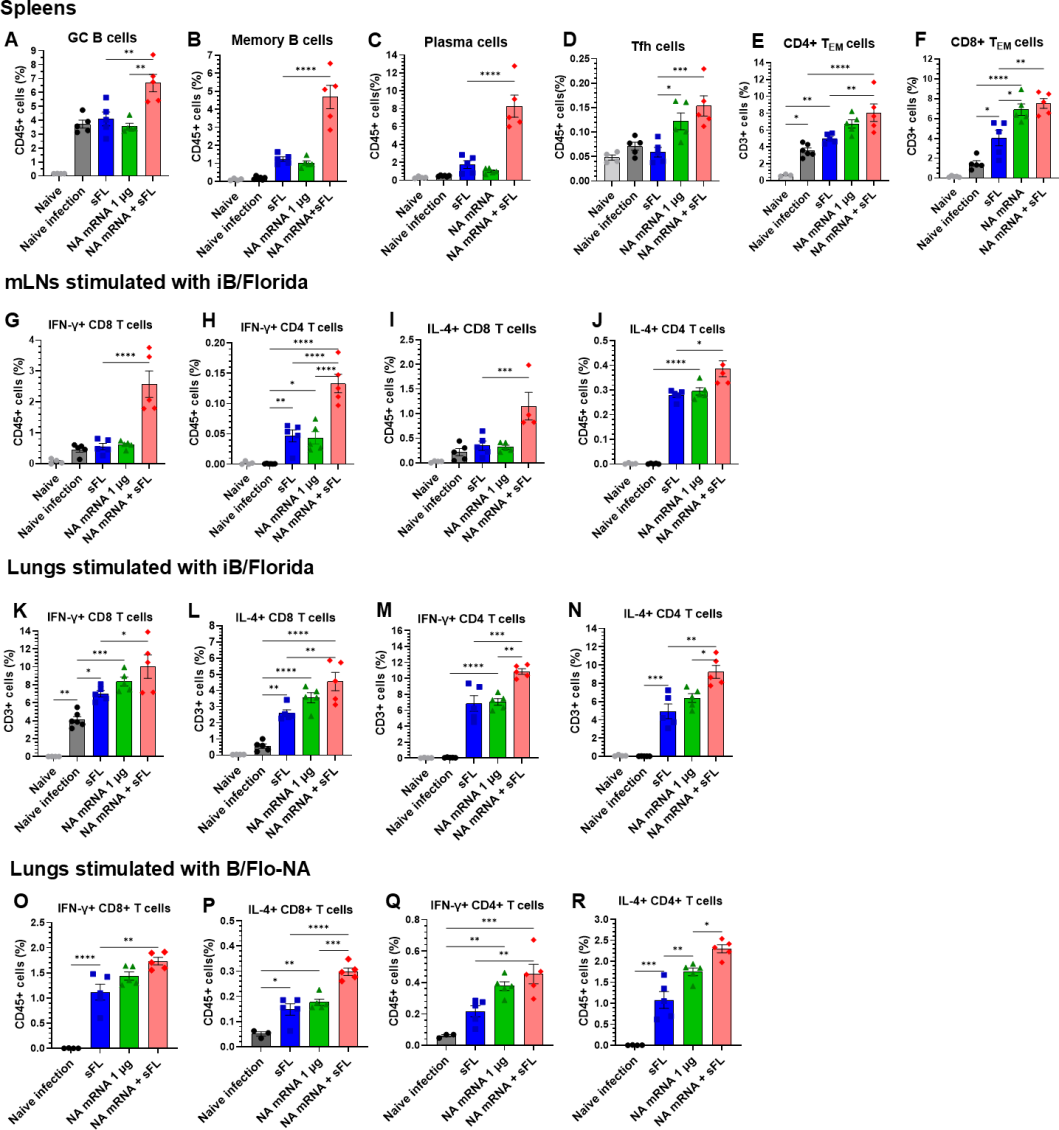
Low-dose NA mRNA + sFL vaccination significantly enhances cellular immune responses. Cellular immune responses were characterized by flow cytometry at 5 dpi with B/Malaysia/2004 virus in the spleens (**A–F**), mLNs (**G–J**), and lungs (**K–R**) from the same vaccine groups as in Fig. 5 (*n* = 5 mice). (**A**) The percentages of germinal center B (GC B) cells: CD45^+^CD3⁻B220^+^CD19^+^CD4⁻GL7^+^. (**B**) memory B (MBCs) cells: CD45^+^CD3⁻IgGD⁻B220⁻CD138^+^. (**C**) plasma cells (PCs): CD45^+^CD3⁻IgGD⁻CD19^+^B220^+^ CD138⁻GL7⁻. (**D**) T follicular helper (Tfh) cells: CD45^+^CD3^+^CD4^+^PD1^+^CXCR5^+^CD25⁻Foxp3⁻, (**E**) Effector memory T (T_EM_) cells in total CD45^+^ splenocytes: CD4 T_EM_: CD3^+^CD8⁻CD4^+^CD62L⁻CD44^+^. (**F**) CD8 T_EM_: CD3^+^CD4⁻CD8^+^CD62L⁻CD44^+^. Effector IFN-γ^+^ CD8 and CD4, and IL-4^+^ CD8 and CD4 T cells upon *in vitro* stimulation with B/Florida/2004 inactivated virus (**G–N**) or NA protein (**O–R**) were analyzed by flow cytometry. All results are presented as mean ± standard error mean (SEM) with individual dots. Statistical analysis was performed using one-way ANOVA and Tukey’s post-multiple comparison tests. *P*-value is significant at *P* < 0.05; *P < 0.0332, **P < 0.0021, ***P < 0.0002, ****P < 0.0001.

Cellular immune responses were determined in draining mediastinal LN (mLN), lungs, and spleens on 5 dpi with B/Malaysia/2004 (Fig. 7). The NA mRNA (1 µg) +sFL group showed the highest level in germinal center (GC) B cells, memory B cells, and plasma cells in spleens (Fig. 7A through C) as analyzed by a multi-color flow gating strategy (Fig. S8). Both NA mRNA + sFL and NA mRNA (1 µg) groups indicated moderate increases in splenic T follicular helper cells (Tfh) and effector memory CD4^+^ and CD8^+^ T_EM_ cells compared to those in the sFL group (Fig. 7D through F).

We determined effector CD4 and CD8 T-cell responses in lungs and mLNs by intracellular cytokine staining flow cytometry (Fig. S9). The combination of NA mRNA and sFL vaccines displayed the highest levels of IFN-γ^+^ CD8 T and IFN-γ^+^ CD4 T cells, IL-4^+^ CD8 T and IL-4^+^ CD4 T cells, upon *in vitro* stimulation of mLN cells with inactivated B/Florida (iB/Florida, Fig. 7G through J) or NA antigen of B/Florida (Fig. S9B through E). When lung cells were stimulated with iB/Florida (Fig. 7K through N) or NA of B/Florida (Fig. 7O through R), the NA mRNA + sFL group showed moderately higher levels of IFN-γ^+^ CD8 T and IFN-γ^+^ CD4 T cells than those from the sFL, NA mRNA 1 µg, and naïve infection control groups (Fig. 7O through R). Both sFL alone and NA mRNA 1 µg alone groups generated similarly low to moderate levels of these effector CD4 and CD8 T-cell responses in mLNs and lungs at 5 dpi (Fig. 7G through R). In summary, co-immunization with NA mRNA and sFL significantly increased GC and memory B cells, plasma cells, and effector T-cell responses, correlating with efficient cross-lineage protection, compared to sFL alone and NA mRNA 1 µg alone vaccination.

### NA mRNA priming and dose-dependent effects on enhancing the immunogenicity of Victoria lineage sML vaccine

**Fig 8:**
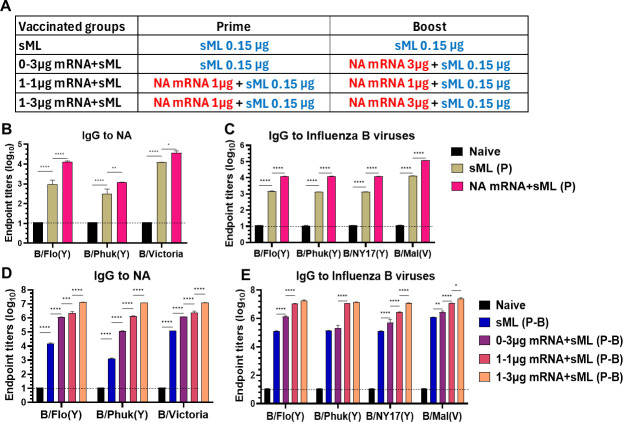
NA mRNA priming and dose effects on enhancing IgG responses to combined sML (**V**) + NA mRNA vaccination. (**A**) Split sML ± mRNA vaccine groups with differential boost doses to determine NA mRNA priming and boost dose effects (*n* = 5 mice). (**B, C**) IgG antibody responses to NA proteins (**B**) and different IBV antigens (**C**) in prime sera. (**D**) IgG antibodies for NA proteins and (**E**) different IBV antigens in boost sera. All results are presented as mean ± standard error mean (SEM) with individual dots. Statistical analysis was performed using two-way ANOVA and Tukey’s post-multiple comparison tests. *P*-value is significant at *P* < 0.05; *P < 0.0332, **P < 0.0021, ***P < 0.0002, ****P < 0.0001.

To extend testing of the adjuvant effects of NA mRNA, we primed the mice with V lineage split vaccine (B/Malaysia/2004, sML 0.15 µg) alone or in combination with 1 µg NA mRNA (Fig. 8A). After prime, the NA mRNA (1 µg) + sML (0.15 µg) group induced IgG antibodies specific for NA (10^4^ titers for NA of B/Florida, 10^3^ titers for NA of B/Phuket, 5 × 10^4^ titers for NA of B/Victoria) at approximately 10-fold higher titers than those in the sML alone group (Fig. 8B). Similarly, NA mRNA + sML prime vaccination increased IgG titers for inactivated IBV antigens (10^4^ titers for B/Flo, B/Phuket, B/NY, 10^5^ titers for B/Mal) by 10-fold compared to sML only prime (Fig. 8C).

Next, we tested the different dosage effects of NA mRNA (0, 1, or 3 µg) in combination with sML during boost vaccinations (Fig. 8A, D, and E). As expected, IgG titers for NA were increased, dependent on prior primed NA mRNA and different boost doses. The 1–3 µg mRNA + sML group showed higher levels of IgG specific for NA (10^7^ titers for NA of B/Flo, B/Phuket, B/Victoria) by 5- to 10-fold than those in the 1–1 µg mRNA + sML and 0–3 µg mRNA + sML groups, which displayed 10 to 100 folds higher IgG titers for NA than sML alone (Fig. 8D). On the other hand, the 1–1 µg and 1–3 µg mRNA + sML groups generated similarly high titers (10^7^) of IgG for cross-lineage IBV antigens (B/Florida, B/Phuket) and homologous B/Mal, whereas 1–3 µg mRNA + sML vaccine showed ~5-fold higher IgG for B/NY17 (Y) than the 1–1 µg mRNA + sML group (Fig. 8E). Higher increases (~100-fold) in IgG titers between the sML and combination (1–3 µg NA mRNA and 1–1 µg NA mRNA but not 0–3 µg NA mRNA) groups were observed for cross-lineage IBV antigens (B/Flo, B/Phuket, B/NY17) than those (~10-fold) for homologous B/Mal (Fig. 8E), suggesting an impact of prior 1 µg NA mRNA priming. The 1–3 µg mRNA + sML group raised M2e specific IgG titers at 20- to 100-fold higher than 1–1 µg mRNA + sML and 100- to 1000-fold higher than 0–3 µg mRNA + sML, suggesting an effect of both prior chimeric NA (M2e-NA) mRNA priming and high-dose boost (Fig. S4B).

### NA mRNA inclusion in prime and boost combination vaccines is important for enhancing functional antibodies and cross-lineage protection

**Fig 9:**
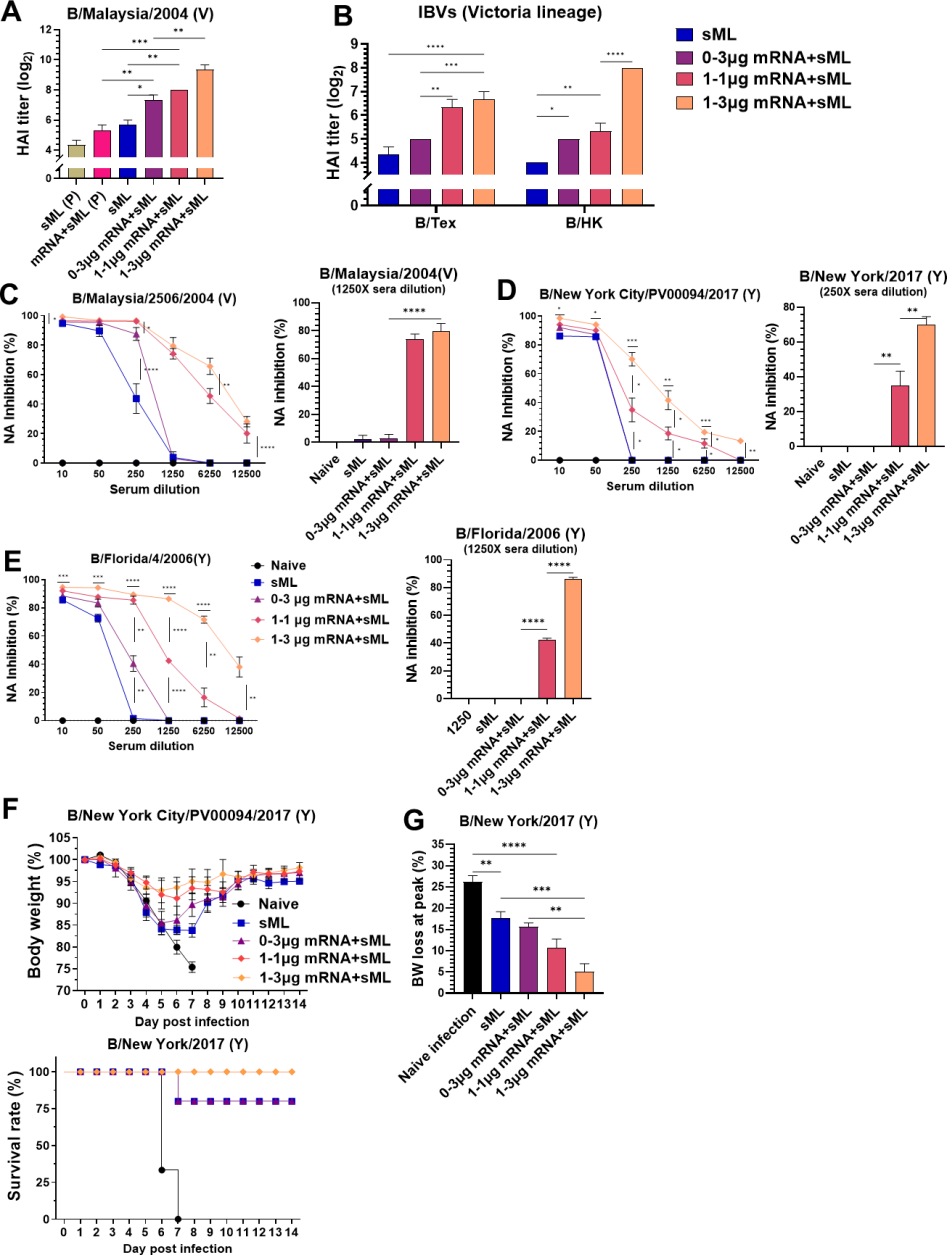
NA mRNA prime and boost dose effects on enhancing cross-reactive humoral immunity and cross-lineage protection after sML (V) + NA mRNA vaccination. (**A**) HAI Titers against B/Malaysia/2004 (V) prime and boost sera. (**B**) HAI titers against B/Texas (V) and B/Hong Kong/2003 (V) in boost sera. (**C, D**) NAI titers in serial dilutions of boost sera and NAI comparisons by bar graphs at a representative dilution. (**C**) NAI against homologous B/Malaysia/2004 (V) virus at serial and 1,250× dilutions. (**D**) NAI activities against B/New York/2017 (Y) virus at serial and 250× dilutions. (**E**) NAI activities against B/Florida/2004 (Y) virus at serial and 1,250× dilutions. (**F**) BW changes and survival rates, and (**G**) comparison of peak weight loss in the differential boost vaccination groups after challenge with cross-lineage B/New York/2017 (Y, 7.9 × 10^3^ EID50) virus. All results are presented as mean ± standard error mean (SEM) with individual mice (*n* = 5). Statistical analysis was performed using one-way ANOVA and Tukey’s post-multiple comparison tests (**A**), two-way ANOVA and Tukey’s post-multiple comparison tests (**B–G**). *P*-value is significant at *P* < 0.05; *P < 0.0332, **P < 0.0021, ***P < 0.0002, ****P < 0.0001.

The NA mRNA (1 µg) + sML prime (P) group increased HAI titers against B/Malaysia/2506/2004 (V) by 2-fold, compared to sML (P) prime sera (Fig. 9A). After boost, the 1–3 µg mRNA + sML group increased HAI titers against homologous (B/Malaysia/2506/2004) by 8-fold (Fig. 9A), and heterologous B/Texas/3/2013 (V) by 5-fold and B/Hong Kong/330/2001 (V) by 16-fold (Fig. 9A and B) compared to those in the sML vaccine. Also, the 1–3 µg mRNA + sML group showed higher titers of HAI against B/Mal by 4-fold and B/HK by 8-fold than the 1–1 µg mRNA + sML group and 0–3 µg mRNA + sML group, supporting the impact of NA mRNA prime and boost dose (Fig. 9A and B). The 1–1 µg mRNA + sML group induced similar HAI titers against B/Texas (V) but lower HAI titers against B/Hong Kong/2001 (B/HK, V) by 5-fold, compared to those in the 1–3 µg mRNA + sML group (Fig. 9B).

NAI activities against homologous virus (B/Malaysia/2004, V) were approximately 25-fold higher in the 1–1 µg mRNA + sML and 1–3 µg mRNA + sML groups than those in the sML alone and 0–3 µg mRNA + sML groups (Fig. 9C). The 1–3 µg mRNA + sML group exhibited NAI activities against cross-lineage viruses B/NY17 (Y) and B/Florida (Y) at 5-fold and 25-fold higher respectively than those from 1 to 1 µg mRNA + sML boost (Fig. 9D and E), suggesting the impact of boost mRNA dose. Nonetheless, 1–1 µg mRNA + sML boost sera showed significantly higher levels of cross-lineage NAI activity than the 0–3 µg mRNA + sML whose NAI is similar (NAI against B/NY17) to or moderately higher (NAI against B/Florida) than sML boost sera (Fig. 9D and E). These results indicate the role of NA mRNA priming in enhancing NAI titers.

To assess the efficacy of cross-lineage protection, the groups of mice with active vaccination were challenged with B/NY/17 (Y) (Fig. 9F and G). Significant weight loss (~15%–18%) and morbidity (75% survival rate) were observed in the 0–3 µg mRNA + sML and sML groups after cross-lineage B/NY/17 (Y) virus challenge (Fig. 9F and G). The 1–3 µg mRNA + sML and 1–1 µg mRNA + sML groups exhibited the least weight loss (~6% and 9%, respectively) against the lethal dose B/NY/17 virus challenge (Fig. 9F and G), correlating with NAI activities against B/NY17 (Fig. 9D). The sFL group displayed 80% survival rates. In contrast, naïve control mice did not survive B/NY/17 virus challenge (Fig. 9G). The naïve mice inoculated with virus and prime sera of split sML (0.15 µg) were protected against a lethal dose of homologous B/Malaysia/04 virus without weight loss (Fig. S10). These results suggest that the inclusion of NA mRNA in prime and mRNA boost doses has a significant impact on improving cross-lineage NAI activity and IBV protection.

## DISCUSSION

NA is the second major glycoprotein and less variable than HA. Immunity to both M2e and NA would provide immunologic benefits in broadening cross-protection against influenza. Here, we designed a new mRNA vaccine construct encoding IBV NA conjugated to tandem repeat 4xM2e. This NA mRNA vaccine raised IgG antibodies cross-reactive to different IBV NA and viral antigens as well as diverse M2e epitopes at 3 µg NA mRNA dose. We found that functional NAI activities against different lineage IBV were induced by NA mRNA 3 µg dose vaccine at varying levels depending on IBV strains. NAI appeared to be correlated with protection against B/Florida (Y), preventing weight loss, probably due to the induction of the highest NAI activities. NA amino acid (aa) homology does not faithfully correlate with NAI titers. Specifically, we observed lower NAI activities against B/NY/2017 (Y) and B/Malaysia (V) than those against B/Florida, although both IBVs have similar (~98.5%) or higher (~99.2%) aa homology with NA mRNA vaccine (Table S1). It is important to note that NA mRNA (3 µg but not 1 µg dose) vaccine could confer significant broad protection against B/Hong Kong (V) and B/Malaysia (V) by preventing severe weight loss, despite low homology (94.6%–96.4%). Meanwhile, NA mRNA at a low dose (1 µg) could induce cross-lineage survival protection with substantial weight loss (13%–14%). The efficacy of broad protection against IBV is consistent with a previous study using a 5 µg dose of monomeric NA (B/Colorado/17, V) mRNA vaccine (13).

Previous studies reported a range of using high doses (5–50 µg) of influenza, COVID-19, and respiratory syncytial virus mRNA vaccines in mice (11, 12, 17, 20). Considering the fact that LNP vehicle for encapsulating mRNA vaccines has an inflammatory nature itself (16, 17), high doses of mRNA LNP vaccines are more likely to cause undesirable side effects in humans (21, 22). A low dose of NA mRNA (1 µg) was less effective in inducing IgG for NA and IBV antigens and NAI activity as well, resulting in lower efficacy of protection against B/Florida (Y) compared to sFL (0.3 µg), which was sufficient for homologous protection via HA neutralizing immunity. Nonetheless, NA mRNA (1 µg) could induce NA-specific IgG at low levels and survival protection against B/Malaysia (V), suggesting that antigen-specific T-cell responses induced by NA mRNA vaccination might have partially contributed to protection against flu B virus. This is due to the non-neutralizing immunity of immune-subdominant universal vaccine targets such as M2e, NA, and HA stalk domains, which often require high doses (10 µg) of protein vaccines or multiple vaccinations (8, 23, 24).

We tested the hypothesis that the combination of low-dose NA mRNA and split vaccines would enhance immunogenicity and cross-lineage protection against IBV. NA mRNA (1 µg) + sFL (0.3 µg) vaccination would enhance the levels of NA-specific IgG antibodies and cross-lineage NAI titers compared to the C-mRNA + sFL, sFL, and NA-mRNA vaccine alone groups, correlating with enhanced cross-lineage protection against B/Malaysia (V). No cross-lineage HAI titers were detected in the vaccinated groups. The results support the immunological advantages of including a low-dose NA mRNA vaccine for enhanced cross-protection in the absence of neutralizing HAI titers. Also, this study suggests a new concept that NA mRNA vaccine could play dual roles in providing survival protection via cross-reactive NA immunity and in enhancing cross-lineage protection by co-administered split vaccine, which has an immunologic advantage over C-mRNA. Also, the inclusion of NA mRNA during prime vaccination was critical for effectively inducing cross-reactive NAI activities and cross-lineage protection as we observed significantly enhanced protection against B/New York/2017 (Y) by 1–3 µg mRNA + sML than 0–3 µg mRNA + sML vaccination.

Influenza virus infection in mice induces over-production of inflammatory cytokines and chemokines, and cellular infiltrates into the lung, resulting in inflammation-mediated tissue damage and severe illness (25). A previous study reported that resident rDCs and AMs are long-lived in lung tissues post-vaccination, as the first guard to take up and present antigens to T cells and to eliminate antigens via phagocytosis, respectively (26). Depletion of AMs has resulted in severe morbidity and impaired viral clearance after influenza virus infection (27). Herein, we observed high levels of rDCs and AMs residing in lung tissues from the NA mRNA + sFL vaccinated mice. Clearance of lung viral loads after NA mRNA + sFL vaccination and challenge appears to be correlated with protection against weight loss, suppressing lung inflammatory responses, and inducing protective rDC and AMs in the lung.

T cells play a role in promoting cross-protection in mice (28) and correlate with better control of virus infection in humans (29). Effector IFN-γ^+^CD4, IFN-γ^+^CD8, IL-4^+^CD4, and IL-4^+^CD8 T cells were induced at higher levels in the lungs and mLN from the NA mRNA + sFL group upon stimulation with IBV or NA antigens than those from the sFL and NA mRNA 1 µg groups. High frequencies of effector memory T cells were found in the spleen from the NA mRNA alone and the NA mRNA + sFL groups. LNP of mRNA vaccines was reported to enhance Tfh and GC B cells, promoting humoral responses (11, 17). Plasma and memory B cells contribute to long-term protection via secreting antibodies upon exposure to pathogens (30). Consistent with these prior studies, we found that combined NA mRNA + sFL vaccination resulted in significantly higher percentages of splenic GC B cells, Tfh, plasma cells, and memory B cells than NA mRNA 1 µg or sFL alone. Together with humoral (IgG, NAI, HAI) responses, these cellular responses might have contributed to enhanced cross-lineage protection induced by a new strategy of combined low-dose mRNA and split vaccines.

LNP vehicle to encapsulate mRNA vaccines was reported to induce inflammatory cytokines (IL-6, IL-1β, IFNs) and chemokines (MCP-1, MIP-1), and recruit inflammatory neutrophils and monocytes and antigen-presenting cells (macrophages, dendritic cells) in mouse models using a high dose (5–30 µg) (16, 17, 31). IL-6 cytokine and ionizable cationic lipid of LNP were shown to play a critical role in adjuvanticity of LNP (at an amount to encapsulate 10–30 µg mRNA), with IL-6 levels remaining elevated for over 24 h (17, 31), which might lead to undesirable side effects (16). Here, the use of low-dose NA mRNA (1 µg) vaccine is expected to induce less inflammatory responses, providing safe vaccination. Different vaccines (mRNA, split, combination) would modulate innate immune profiles differentially. Further studies are required to elucidate the underlying innate and adaptive immune mechanisms by which the combination of low-dose NA mRNA + split vaccination induces enhanced cross-protection.

Taken together, the combination of low-dose NA mRNA and split vaccines can provide immunological benefits, including enhanced cross-reactive IgG and functional antibodies (NAI, HAI), contributing to higher efficacy of cross-protection, which is beyond the control mRNA LNP adjuvant effects. Also, the combination of low-dose NA mRNA + split vaccines would induce cellular immune profiles at higher levels than mRNA LNP and split vaccine alone. This study provides a proof-of-concept for the combined low-dose mRNA and conventional vaccines to overcome the limitations of each vaccine platform.

## MATERIALS AND METHODS

### Viruses, mRNA, and split vaccines

Live virus Yamagata (Y) lineage B/Florida/4/2006 (B/Flo, Y), B/New York City/PV00094/2017 (B/NY/2017, Y), Victoria (V) lineage B/Malaysia/2506/2004 (B/Mal, V) and B/Hong Kong/330/2001 (B/HK, V) were used for virus challenges. Live viruses B/Phuket/3037/2013 (B/Phuk, Y), B/Texas/2/2013 (B/Tex, V), and reassortant A/Nanchang/933/1995 H3N2 (A/Nanchang/H3N2) were amplified by propagation in 10-day-old embryonated chicken eggs. The gene for NA mRNA vaccine translating tPA-4xM2e-Tetrabrachion-consensus B NA ectodomain (NA mRNA, Fig. 1A; Fig. S1) was codon-optimized by using a GenSmart Optimization Tool and then further optimized by increasing G/C contents and minimizing uridine usage. The optimized open reading frame gene was cloned into mRNA transcription plasmid via GenScript customer service. Uridine residues were replaced by N1-methyl pseudouridines during *in vitro* transcription, together with 5′-untranslated region (UTR) and 3′-UTR modified from the sequence reported (32) as well as CleanCap1 and poly-A additions. *In vitro* transcribed mRNA products were purified by silica membrane and dsRNA removal (GenScript). Similarly, we prepared SARS-CoV-2 spike ectodomain encoding mRNA which was used as control mRNA (C-mRNA) via TriLink customer service. Enhanced green fluorescent protein (eGFP) mRNA (N1-Methyl-Ψ) was purchased (GenScript). Inactivated split vaccine (sFL, sML) strains used to immunize mice were B/Florida/4/2006 (Y) for sFL and B/Malaysia/2506/2004 (V) for sML, which were produced by 1% formalin-inactivation, followed by Triton X-100 treatment to disrupt virion particles as described (33). Hemagglutination activity units (HAU) assay was used to determine the quality of the split vaccines.

### Cell transfections and reagents

Human Embryonic Kidney (HEK) 293T (CRL-3216, ATCC) cells and Madin–Darby canine kidney (MDCK) (CCL-34, ATCC) were cultured in Dulbecco’s modified Eagle’s medium (DMEM) supplemented with 10% Fetal Bovine Serum (FBS) (Gibco), 100 U/mL penicillin, and 100 mg/mL streptomycin (Gibco). HEK 293T cells were seeded in a 96-well plate at a concentration of 10^4^ cells/well in complete medium [DMEM supplemented 10% fetal bovine serum (FBS) and penicillin (100 U/mL)-streptomycin (100 µg/mL) solution] and transfected by Lipofectamine 2000 Transfection Reagent (ThermoFisher Scientific). mRNA (0.1 µg) in opti-MEM was mixed with Lipofectamine in opti-MEM, and the complex was added to cultured cells in a complete medium. IBV NA proteins were obtained from BEI resources (NA of B/Florida/4/2006) or purchased from Sino Biological (NA of B/Phuket/3073/2013, B/Victoria/705/2018).

### mRNA encapsulation with LNP

Optimized LNP mixtures (GenVoy-ILM) commercially available from Cytiva contain PNI ionizable cationic lipid, DSPC (1,2-distearoyl-sn-glycero-3-phosphocholine), PEG-lipids (PNI stabilizer), and cholesterol. We prepared mRNA-LNPs (mRNA vaccine) by NanoAssemblr Benchtop Instrument (Precision NanoSystems), which enables rapid fluidic mixing and encapsulating mRNA in LNPs (34). A RoboGreen assay was used to determine the content of mRNA in encapsulated LNPs by following the Encapsulation Efficiency Assay Protocol for RNA-LNPs (Precision NanoSystems).

### Immunofluorescence assay and IgG cell ELISA

eGFP mRNA or NA mRNA-transfected HEK 293T cells 24 h later were fixed with 100 µL of 4% formaldehyde for 15 min at room temperature. Cells were washed three times with PT buffer [0.3% Triton 100X in 1X phosphate-buffered saline (PBS)], and then blocked by PBST buffer with 4% bovine serum albumin. Next, cells were incubated with anti-M2e monoclonal antibody (14C2 mAb, Abcam Inc., Cambridge, MA, USA), or NA polyclonal antisera at 4℃. Cells were washed with PT buffer and incubated with goat anti-mouse IgG-conjugated AlexaFluor-488 (ThermoFisher Scientific). After washing, the cells were examined and photographed under the fluorescence microscope. For cell ELISA, the washed cells were incubated with horseradish peroxidase (HRP)-conjugated anti-mouse IgG antibody for 90 min in the dark, followed by Tetramethylbenzidine (TMB) used as a substrate. Absorbance was obtained at an optical density (OD) of 450 nm by using an ELISA reader.

### Mice and immunization

BALB/c mice (6–8 weeks old, females) were purchased from Jackson Laboratories and housed at an animal facility at GSU. Mice were intramuscularly (i.m.) immunized at two times (4-week interval) with 100 µL of NA mRNA-LNP alone (1 µg or 3 µg), inactivated split vaccines (0.3 µg sFL) ± mRNA LNP (1 µg of NA mRNA-LNP or C-mRNA-LNP). For the second set of experiments, mice were i.m. immunized with a prime dose of 100 µL of sML (0.15 µg) ± NA mRNA-LNP (1 µg), and a second dose of sML (0.15 µg) ± NA mRNA-LNP (0, 1, or 3 µg). Immunized mice and naïve mice were then challenged with a lethal dose of B/Flo (Y, 1.07–1.43 × 10⁵ EID_50_), B/NY2017 (Y, 7.9 × 10³ EID_50_), B/Mal (V, 1.58 × 10⁵ EID_50_), or B/HK (V, 1.4 × 10⁵ EID_50_) at 4 weeks after boost dose. The mice’s body weight (BW) was then monitored daily for 14 days. The mice displaying more than 20% BW loss were considered to have reached the endpoint and humanely euthanized to minimize pain.

### ELISA antibody and cytokine productions

Immune sera were collected at 2 weeks after each immunization to determine antigen-specific IgG antibody levels. ELISA plates were coated with inactivated B/Flo, B/Phuket/3073/2017 (B/Phuk), B/NY2017, B/Mal, and M2e peptides at a concentration of 200 ng/well; NA (from B/Flo, B/Phuk, B/Victoria) at a concentration of 20 ng/well. The HRP-labeled secondary antibodies were used to detect antigen-specific IgG antibodies. TMB was used as a substrate, and absorbance was obtained at an OD of 450 nm by using an ELISA reader. IgG titers were determined as the maximum dilutions giving OD values above the naïve serum control.

The levels of cytokines and chemokines in lung extracts obtained from immunized mice post-challenge were measured using interleukin (IL)-1β, IL-6, IL-4, IL-12p70, interferon IFN-γ and tumor necrosis factor (TNF)-α kits from Invitrogen (Waltham, MA, USA), and monocyte chemoattractant protein-1 (CCL2/MCP-1), C–C motif chemokine ligand 5 (CCL5), C–X–C motif chemokine ligand 10 (CXCL-10), and C–X–C chemokine ligand 1 (CXCL-1) kits from R&D Systems (Minneapolis, MN, USA) in accordance with the manufacturers’ instructions.

### Neuraminidase inhibition assay

Flat-bottom 96-well plate was coated with fetuin (Sigma) at 25 µg/mL in PBS. Fetuin-coated plate was then blocked with 1% BSA in PBST. B/Florida/2006, B/Phuket/2013, B/New York/2017, and B/Malaysia/2004 IBVs were serially diluted 2-fold or 5-fold and added to the fetuin-coated plates for incubation as a positive control. To measure NAI, boost immunized sera were inactivated at 56°C for 30 min, and then diluted serially 2-fold in sample diluent with a starting dilution of 1:10. Diluted sera were mixed with an equal volume of respective diluted viruses and incubated for 35 min. The mixer (50 µL) of diluted sera and viruses was added to the fetuin-coated plate and incubated for 18 h at 33°C. HRP-conjugated peanut agglutinin in PBS was added to the plates and incubated. TMB was used as a substrate to develop the color, and absorbance was obtained at an OD of 450 nm.

### Hemagglutination inhibition assay

Sera collected from immunized BALB/c mice were inactivated by incubation at 56°C for 30 min and then treated with the receptor-destroying enzyme (RDE, Sigma-Aldrich) for 18 h at 37°C. Subsequently, sera were serially diluted in PBS and incubated with 4 HA units of B/Flo, B/Phuk, B/NY2017, or B/Mal for 30 min, and then 0.5% chicken red blood cells were added to determine HAI titers.

### Lung viral titration

Lungs of the infected BALB/c mice were harvested at 5 dpi and minced mechanically with 1.5 mL of Roswell Park Memorial Institute (RPMI) 1640 medium (Fisher Scientific, Corning, NY, USA). Lung extracts were collected by centrifugation for viral titration. 10-day-old embryonated chicken eggs were inoculated with serially diluted lung extracts, and viral titers were determined by HA assay of the allantoic fluids collected after 3 days. Viral titers at 50% egg infection dose (EID_50_)/mL were evaluated using the Reed and Muench method (35).

### Flow cytometry

Inflammatory cell infiltrations were analyzed by collecting lung samples at 5 dpi and staining single cells with antibodies specific for CD45 (clone 30-F11), CD3 (clone 17A2), CD11b (clone M1/70), CD11c (clone N418), F4/80 (clone BM8), Ly6c (clone AL-21), major histocompatibility complex (MHC) class II (clone I-A/I-E), SiglecF (clone E50-2440), B220 (RA3-6B2), and live/dead-amcyan (LIVE/DEAD Fixable Aqua Dead Cell Stain Kit) after blocking the Fc receptor by anti-CD16/CD32 monoclonal antibody (mAb). Spleens were harvested, homogenized to single cells, and stained with antibodies specific for CD45, CD3, CD4 (clone RM4.5), CD8a (clone 53–6.7), CD44 (clone IM7), and CD62L (clone MEL-14) to determine the memory phenotypic T cells. Splenocytes were stained with antibodies specific for CD45, CD3, CD4, IgD (clone 11.26), B220, CD19 (clone eBio1D3), CD138 (clone 281–2), GL7 (clone GL7), PD-1 (clone eBioJ105), CXCR5 (clone L138D7), CD25 (clone PC61.5), Foxp3 (clone FJK-16s), and live/dead cells to investigate germinal center (GC) B cells, memory B cells, and Tfh cells. Flow cytometry gating strategies are shown in Fig. S7 to S9.

Intracellular cytokines [IL-4 (clone 11B11) and IFN-γ (clone XMG1.2)] of CD4 and CD8 T cells residing in the lungs and lymph nodes were stained after 5 h stimulation with inactivated B/Florida/2006 or NA (B/Flo) protein to estimate the antigen-specific IL-4/IFN-γ secreting CD4 and CD8 T cells. Golgi stop (monensin, a protein transport inhibitor) was added to each sample for *in vitro* stimulation. Intracellular cytokine staining was performed using the fixation/permeabilization solution kit (BD Biosciences) in accordance with the manufacturer’s instructions.

### Statistical analysis

All the results are presented as the mean ± standard error of the mean (SEM). Statistical significance was determined using one-way ANOVA and Tukey’s post-multiple comparison tests, two-way ANOVA and Tukey’s post-multiple comparison tests. *P*-value is considered at *P* < 0.05; *P < 0.0332, **P < 0.0021, ***P < 0.0002, ****P < 0.0001. All data were analyzed using GraphPad Prism 9 statistical software Inc. (San Diego, CA, USA).

Experiments were conducted with sufficient power (*n* = 3–10 mice/group) to ensure distinct differences between the groups to be detected.

## Data Availability

All relevant data generated during this study are available upon reasonable request made to the corresponding author. The underlying code for this study is not publicly available for proprietary reasons.

## SUPPLEMENTAL MATERIAL

Supplemental figures jvi.02294-24-s0001.pdf Table S1; Figures S1 to S10.

ASM does not own the copyrights to Supplemental Material that may be linked to, or accessed through, an article. The authors have granted ASM a non-exclusive, world-wide license to publish the Supplemental Material files. Please contact the corresponding author directly for reuse.
