# Supplementary material for: Dual roles of influenza B virus neuraminidase mRNA vaccine in enhancing cross-lineage protection by supplementing inactivated split vaccination
Source: J Virol. 2025 Apr 23;99(5):e02294-24. doi: 10.1128/jvi.02294-24 (PMC12090766; doi:10.1128/jvi.02294-24)
Supplement: Supplemental figures — Table S1; Figures S1 to S10. [file jvi.02294-24-s0001.pdf]

Supplementary Table S1. Amino acid similarity (%) between vaccine antigens and challenge virus strains.

Viruses	Vaccine antigens		
	**B.NA mRNA**	**Split B/Flo HA**	**Split B/Mal HA**
B/Florida/4/2006 (Y)	98.71	100	93.12
B/New York City/PV00094/2017 (Y)	98.46	97.95	92.77
B/Phuket/3073/2013 (Y)	99.23	98.29	92.77
B/Hong Kong/330/2001 (V)	94.60	93.68	98.28
B/Malaysia/2506/2004 (V)	96.40	93.12	100
B/Texas/02/2013 (V)	95.28	93.33	98.8

Amino acid sequences from B NA mRNA, inactivated split B/Florida/2006 (sFL), or inactivated split B/Malaysia/2004 (sML) vaccines were aligned with the amino acid sequences of hemagglutinin (HA) and neuraminidase (NA) from influenza virus strains in this study (B/Florida/4/2006 (Y), B/New York City/PV00094/2017 (Y), B/Phuket/3073/2013 (Y), B/Hong Kong/330/2001 (V), B/Malaysia/2056/2004 (V), and B/Texas/02/2013 (V)) using Clustal Omega Multiple Sequence Alignment tool. The percentages of amino acid sequence similarities (%) were determined using EMBOSS Matcher Pairwise Sequence Alignment.

Chimeric NA mRNA vaccine construct:
tPA (SP) – 4xM2e –tetramer stabilizing domain- consensus NA ectodomain full length
[Globular head domain aa 82-471]

```
MDAMKRGLCCVLLLCGAVFVSASQE          tPA signal peptide
SLLTEVETPIRNEWGSRSNDSSDGGSGGG      hM2e-L1 linker
SLLTEVETPTRSEWESRSSDSSDAAAPGAA     sM2e-L2 linker
SLLTEVETPTRNEWESRSSDSSDAAGGGA      a1M2e-L3 linker
SLLTEVETPTRTGWESNSNGSSDAAPGGSG     a2M2e-L4 linker
IINETADDIVYRLTVIIDDRYESLKNLITLRADRLEMIINDNVSTILASGGSGG Tetrabrachion-L5 linker
PEWTYPRLSCPGSTFQKALLISPHRFGETKGNSAPLIIREPFI
ACGPKECKHFALTHYAAQPGGYYNGTREDRNKLRHLISVKLGKIPTVENSIFHMAAWSGS
ACHDGKEWTYIGVDGPDSNALLKIKYGEAYTDTYHSYAKNILRTQESACNCIGGDCYLMI
TDGPASGVSECRFLKIREGRIIKEIFPTGRVKHTEECTCGFASNKTIECACRDNSYTAKR
PFVKLNVETDTAEIRLMCTKTYLDTPRPNDGSITGPCESDGDKGSGGIKGGFVHQRMASK
IGRWYSRTMSKTKRMGMGLYVKYDGDPWTDSEALALSGVMVSMEEPGWYSFGFEIKDKKC
DVPCIGIEMVHDGGKTTWHSAATAIYCLMGSGQLLWDTVTGVNMTL**   B NA ectodomain: 82-471 amino
                                                   acid residues
```

Supplementary Figure S1. The amino acid sequence of chimeric B NA mRNA construct. Influenza B virus chimeric NA mRNA vaccine construct encodes tPA (tissue plasminogen activator) signal peptide (SP), a tandem repeat of heterologous M2e and linkers, tetrabrachion (a tetramer stabilizing domain), and NA full-length ectodomain (amino acids 82-471). Each domain is indicated. .

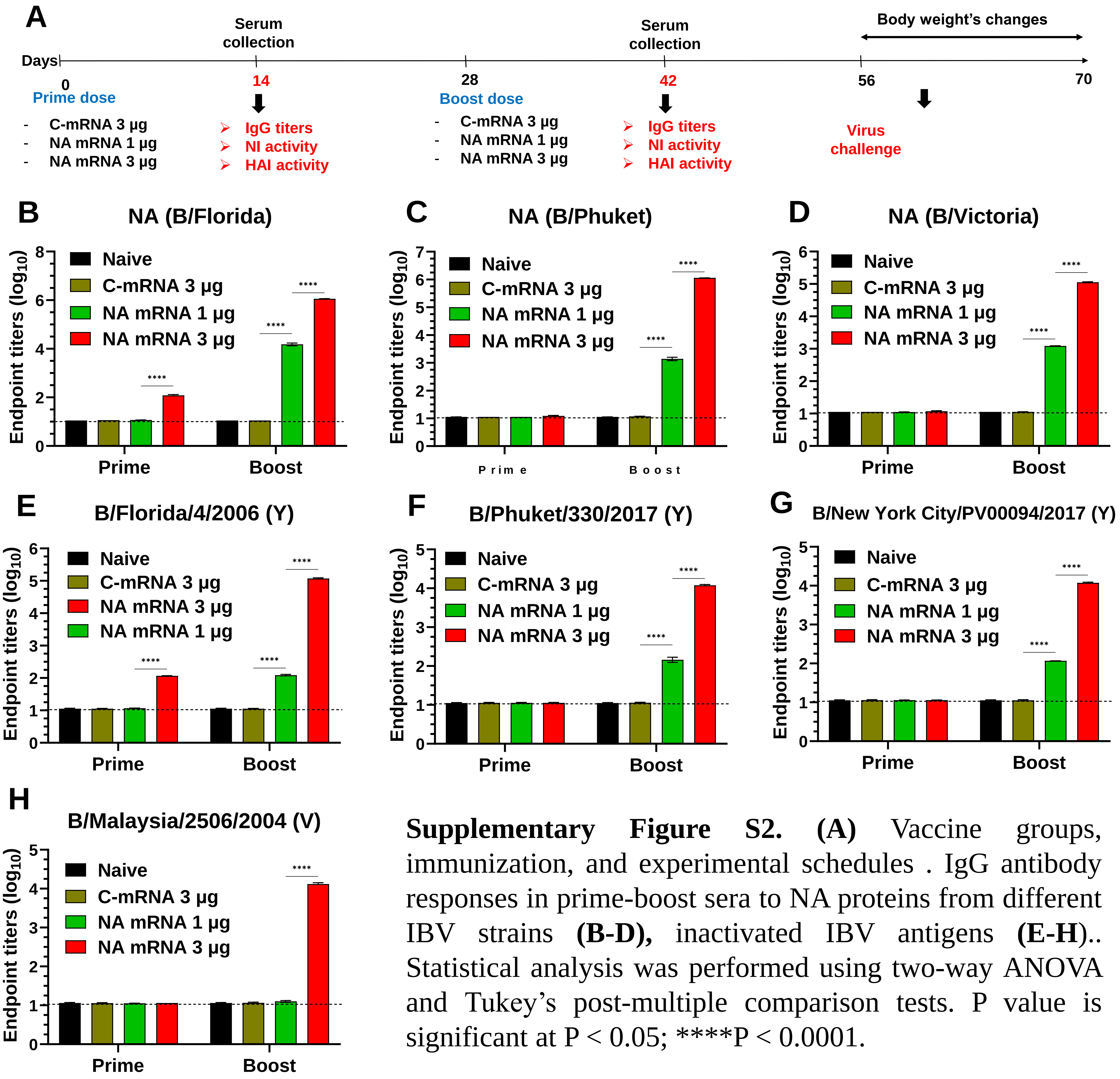

Supplementary Figure S2. **(A)** Vaccine groups, immunization, and experimental schedules . IgG antibody responses in prime-boost sera to NA proteins from different IBV strains **(B-D),** inactivated IBV antigens **(E-H)**.. Statistical analysis was performed using two-way ANOVA and Tukey's post-multiple comparison tests. P value is significant at P < 0.05; ****P < 0.0001.

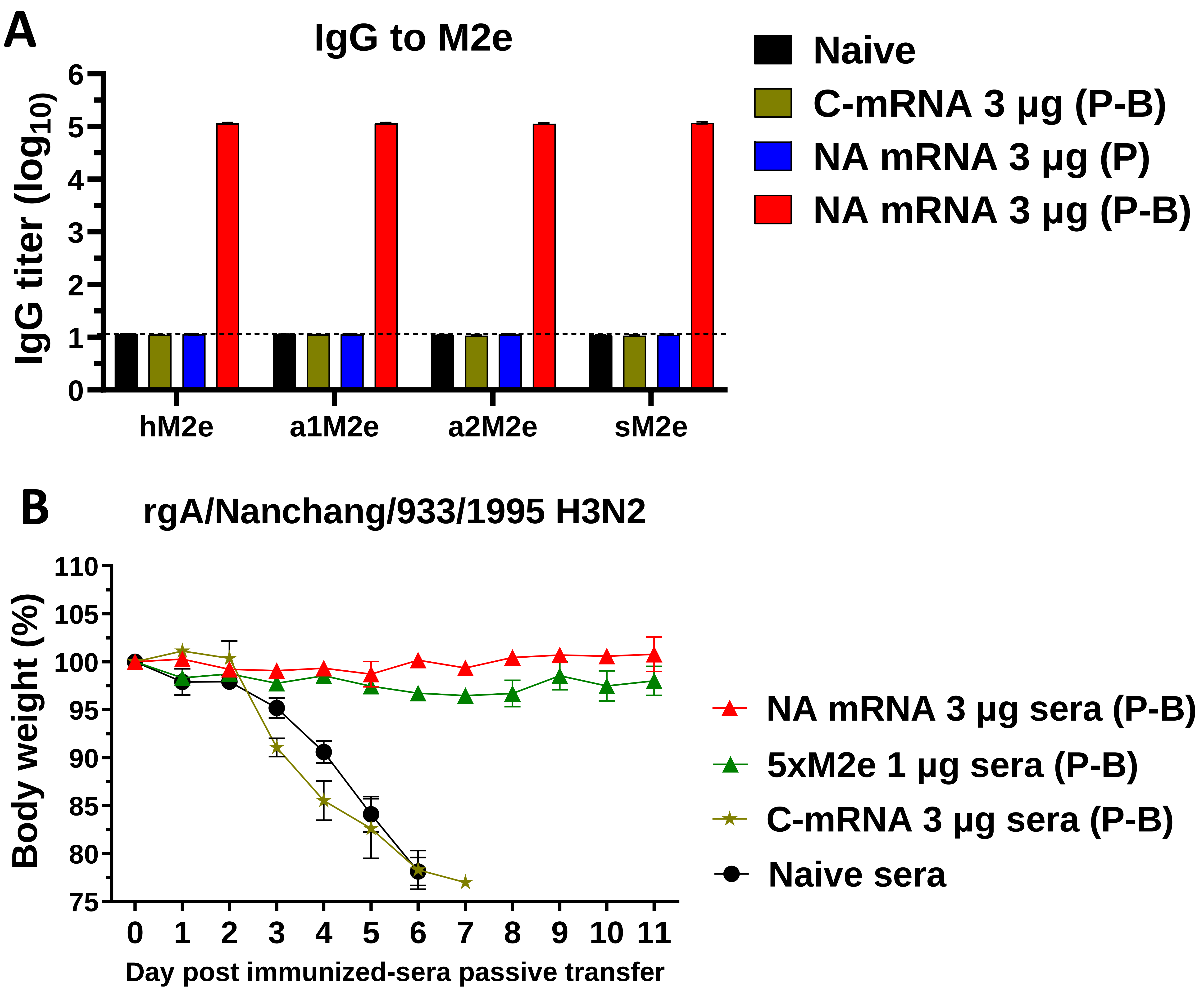

Supplementary Figure S3. (A) IgG antibody responses (P: prime sera, P-B: prime-boost sera) to M2e peptides (human hM2e, avian a1M2e, A/Shanghai H7N9 a2M2e, swine sM2e). Influenza A M2e peptides were synthesized by GenScript: human M2e (hM2e, SLLTEVETPIRNEWGSRSN), swine M2e (sM2e, SLLTEVETPTRSEWESRSS), avian M2e (a1M2e, SLLTEVETPTRNEWESRSS) and influenza A/Shanghai/1/2013 H7N9 M2e (a2M2e, SLLTEVETPTRTGWESNSN). **(B)** *In vivo* protection in naïve mice by antisera of boost vaccination: Naïve mice (n= 3) were intranasally inoculated with a mixture of boost sera or naïve sera (4X dilutions) and rgA/Nanchang/933/1995 H3N2 (3.17 x 10^6 EID50) virus. Mice's body weight changes after (i.n.) inoculation of naïve mice with the mixer of immunized sera with rgA/Nanchang H3N2 virus were daily monitored. NA mRNA: chimeric flu B NA – 4xM2e. M2e repeat only mRNA vaccination sera (No NA component). C-mRNA: Control (SARS CoV-2 spike) mRNA.

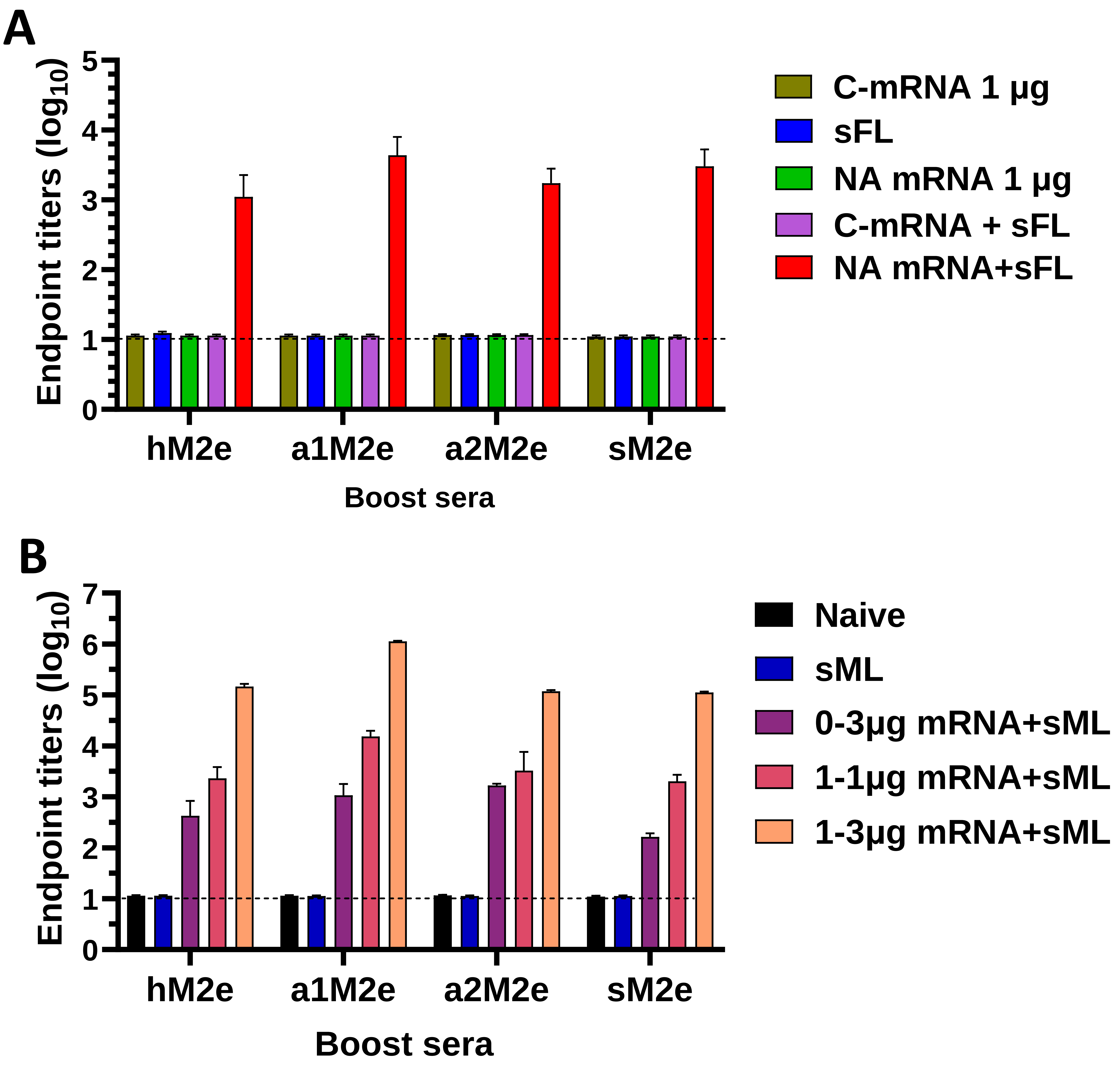

Supplementary Figure S4. Boost serum IgG antibody responses. Boost sera were harvested from immunized mice as described in Figure 3A and 8A, respectively. **(A-B)** IgG titers specific for different M2e peptides (human hM2e, avian a1M2e, A/Shanghai H7N9 a2M2e, swine sM2e).

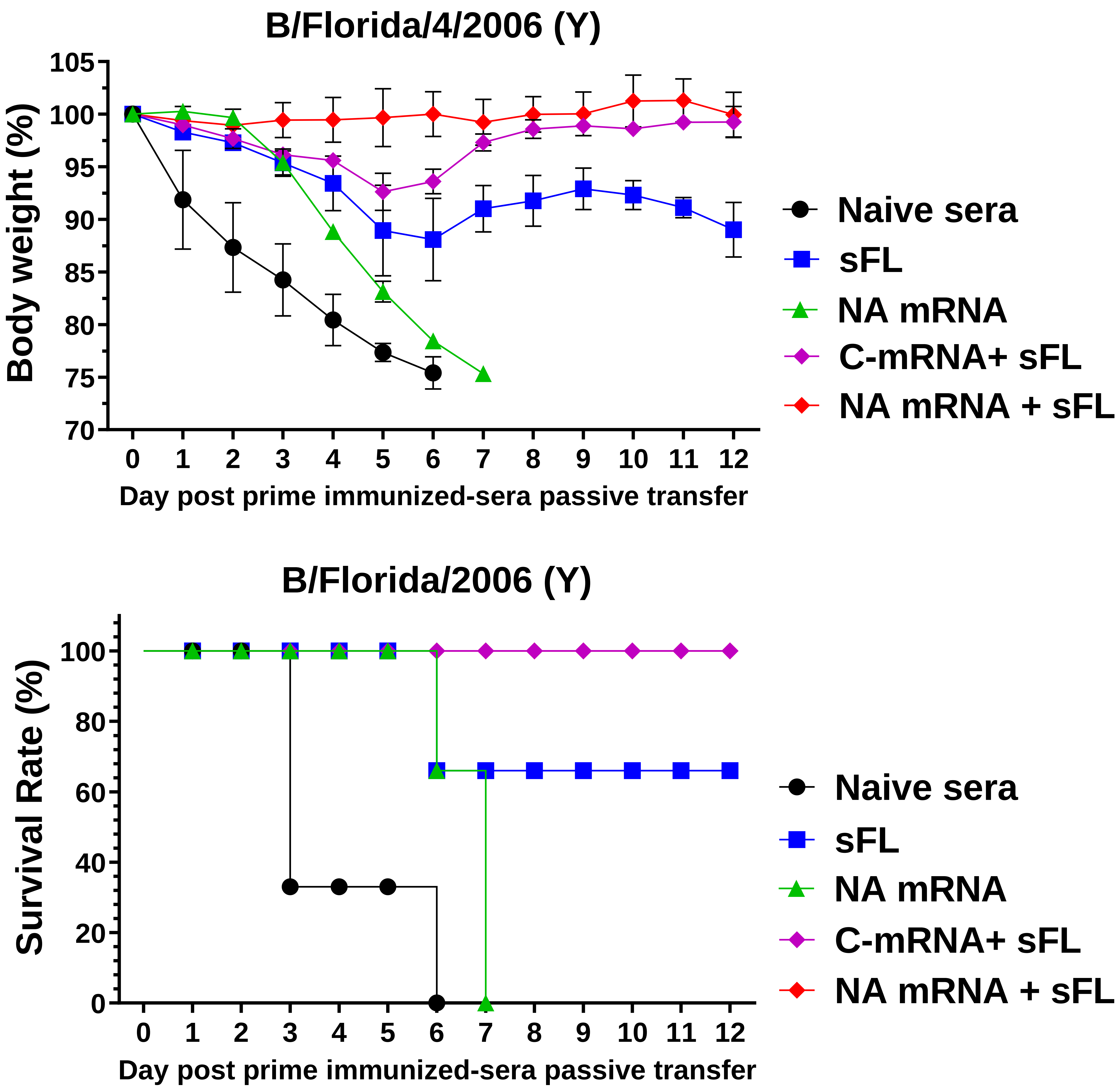

Supplementary Figure S5. Prime antisera confer protection against homologous virus in naïve mice Weight changes and survival rates in naïve BALB/c mice (n=3-4 per group) after intranasal inoculation with a mixture of homologous B/Florida/2006 (Y) virus and prime sera from the sFL (0.3 µg), NA mRNA (1 µg), C-mRNA (1 µg) + sFL (0.3 µg) or NA mRNA (1 µg) + sFl (0.3 µg) vaccinated groups (as described in Figure 3A).

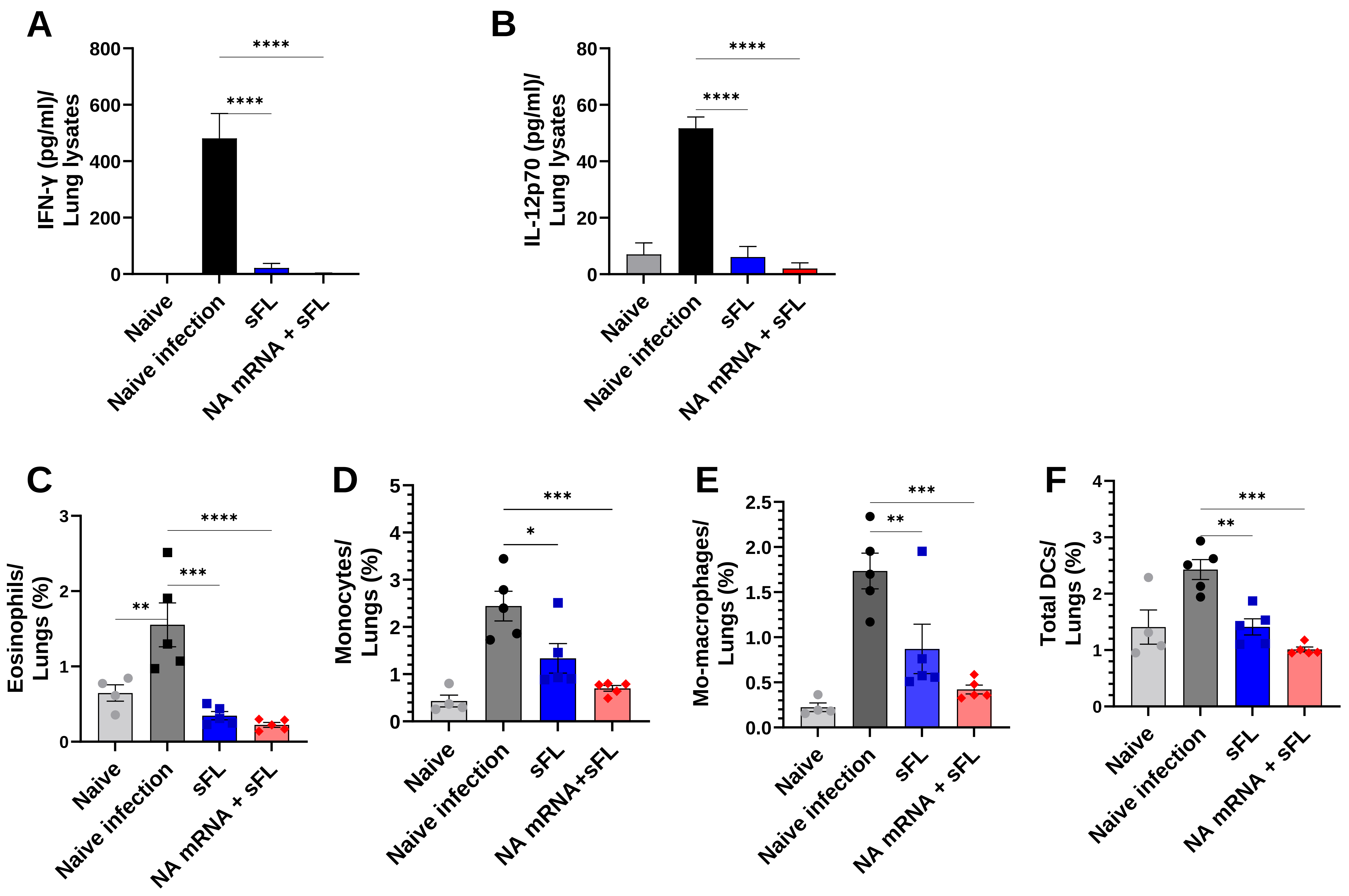

Supplementary Figure S6. Lung inflammatory responses at day 5 post challenge with cross-lineage B/Malaysia/2506/2004 virus. Cytokines and chemokines released in lung extracts **(A, B)** were measured by ELISA. Inflammatory cellular infiltrates into the lungs were assessed by flow cytometry **(C-F)**. **(C)** Eosinophils: $CD45^{+}CD11b^{+}SiglecF^{+}$; **(D)** monocytes: $CD45^{+}F4/80^{-}CD11b^{+}Ly6c^{high}$; **(E)** mo-macrophages: $CD45^{+}$ $F4/80^{+}CD11b^{-}CD11c^{+}$; **(F)** total DCs: $CD45^{+}F4/80^{-}CD11c^{+}MHC\ II^{high}$. All results are presented as mean ± standard error mean (SEM) with individual dots. Statistical analysis was performed using one-way ANOVA and Tukey's post-multiple comparison tests. P value is significant at $P < 0.05$; *$P < 0.0332$, **$P < 0.0021$, ***$P < 0.0002$, ****$P < 0.0001$.

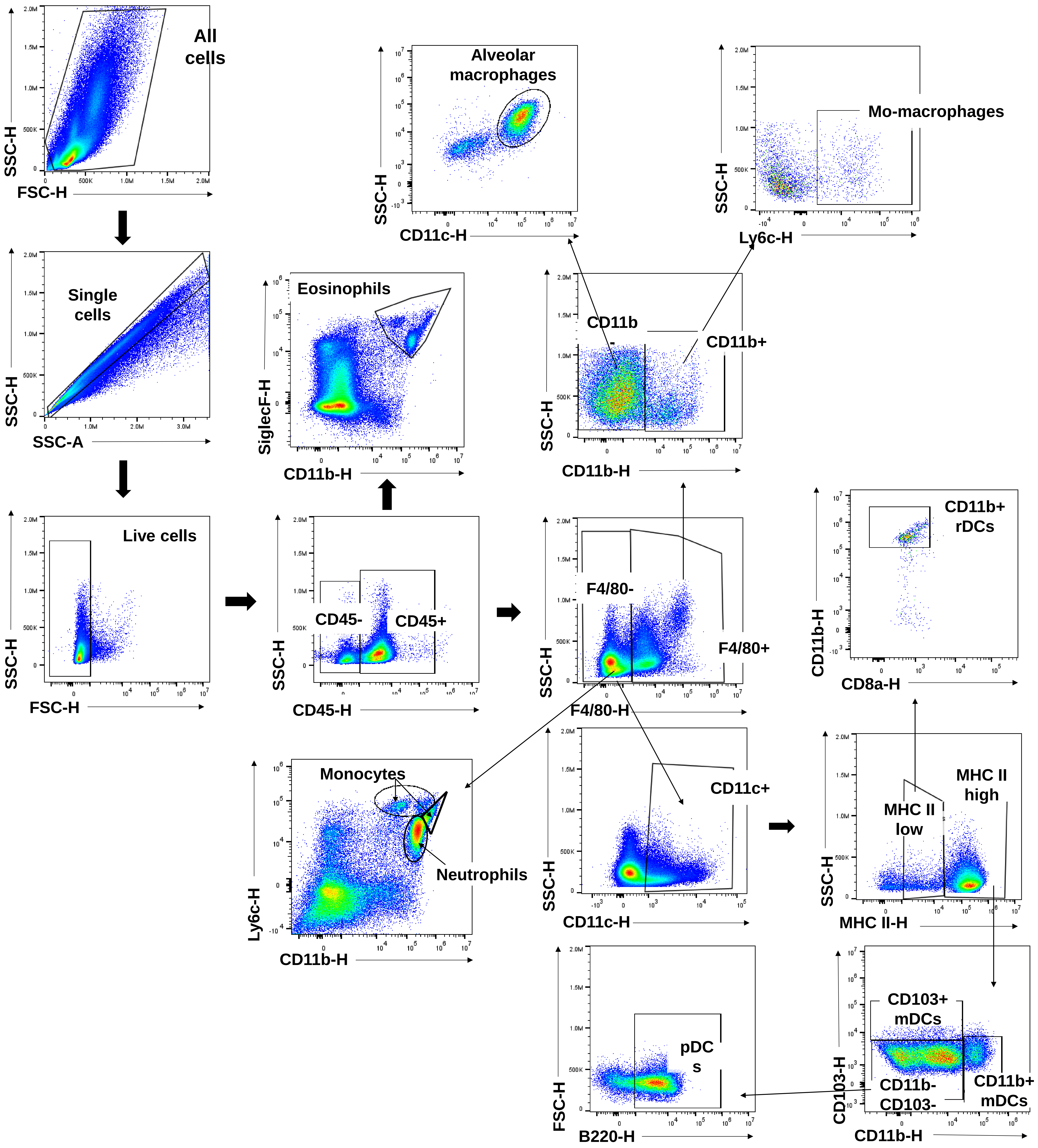

Supplementary Figure S7: Flow cytometry gating strategies for innate immune cells in lung. Frequencies of **Eosinophils**: CD45^{+}CD11b^{+}SiglecF^{+}, **Neutrophils**: CD45^{+}F4/80^{-}CD11b^{+}Ly6c^{low}; **monocytes**: CD45^{+} F4/80^{-} CD11b^{+}Ly6c^{high}; **Plasmacytoid DCs (pDCs)**: CD45^{+}F4/80^{-}CD11c^{+}MHC II^{high}B220^{+}; **CD11b^{+}mDCs**: CD45^{+}F4/80^{-}CD11c^{+}MHC II^{high}CD103^{-}CD11b^{+}; **CD103^{+}mDCs**: CD45^{+}F4/80^{-}CD11c^{+}MHC II^{high}CD103^{+}CD11b^{-}; **CD11b+ resident DCs (rDCs)**: CD45^{+}F4/80^{-}CD11c^{+}MHC II^{low} CD8a^{-}CD11b^{+}; **Monocyte-derived macrophages**: CD45^{+}F4/80^{+}CD11b^{+}Ly6c^{+}, **Alveolar macrophages (AMs)**: CD45^{+}F4/80^{+}CD11b^{-}CD11c^{+}. .

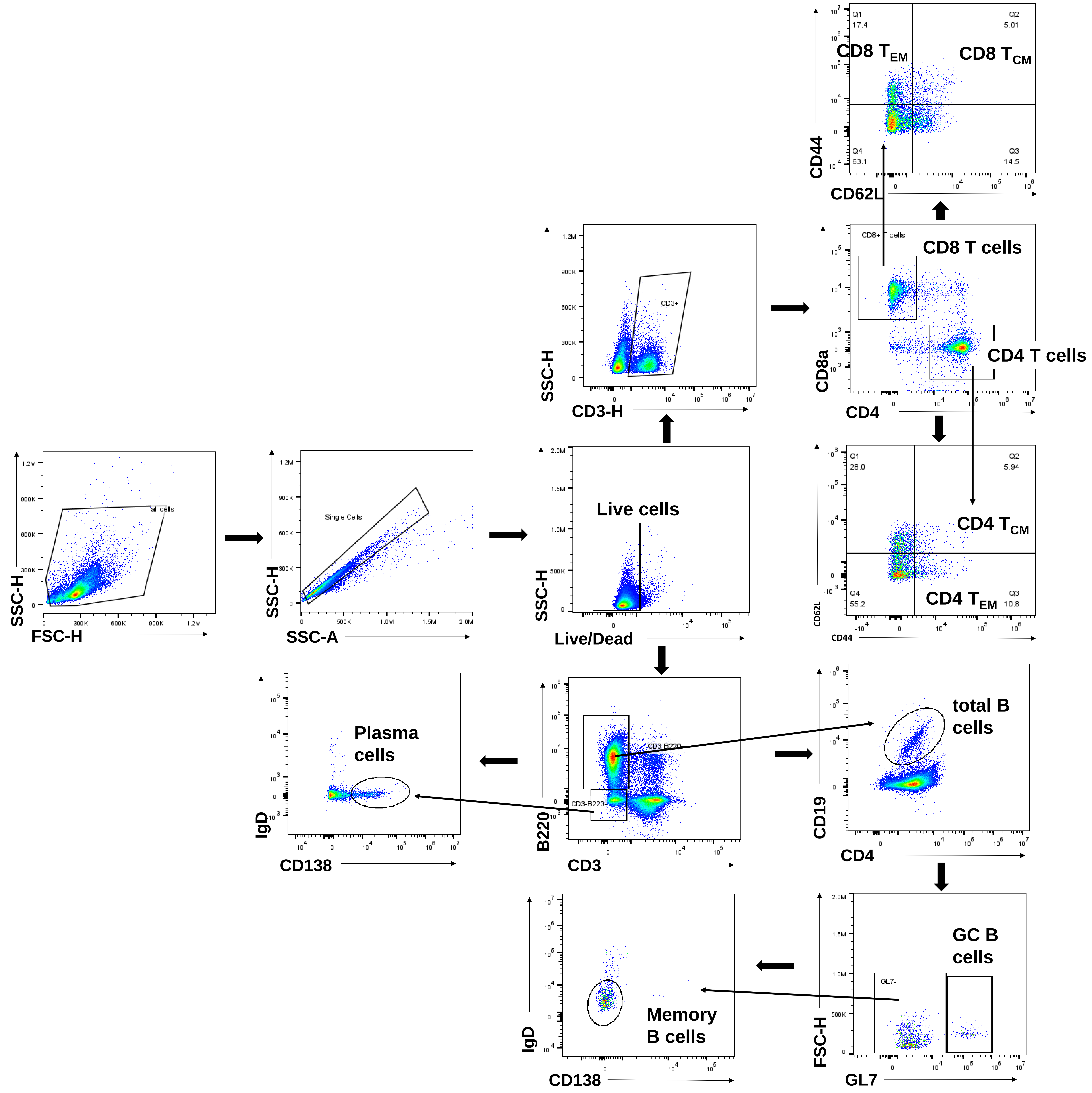

Supplementary Figure S8: Flow cytometry gating strategies for memory T cells and memory B cells. Frequencies of **central memory T (T$_{CM}$)** cells in spleens: **CD4 T$_{CM}$**: CD3$^+$CD8$^-$CD4$^+$CD62L$^+$CD44$^+$, and **CD8 T$_{CM}$**: CD3$^+$CD4$^-$CD8$^+$CD62L$^+$CD44$^+$. **CD4 T$_{EM}$**: CD3$^+$CD8$^-$CD4$^+$CD62L$^-$CD44$^+$, and **CD8 T$_{EM}$**: CD3$^+$CD4$^-$CD8$^+$CD62L$^-$CD44$^+$.

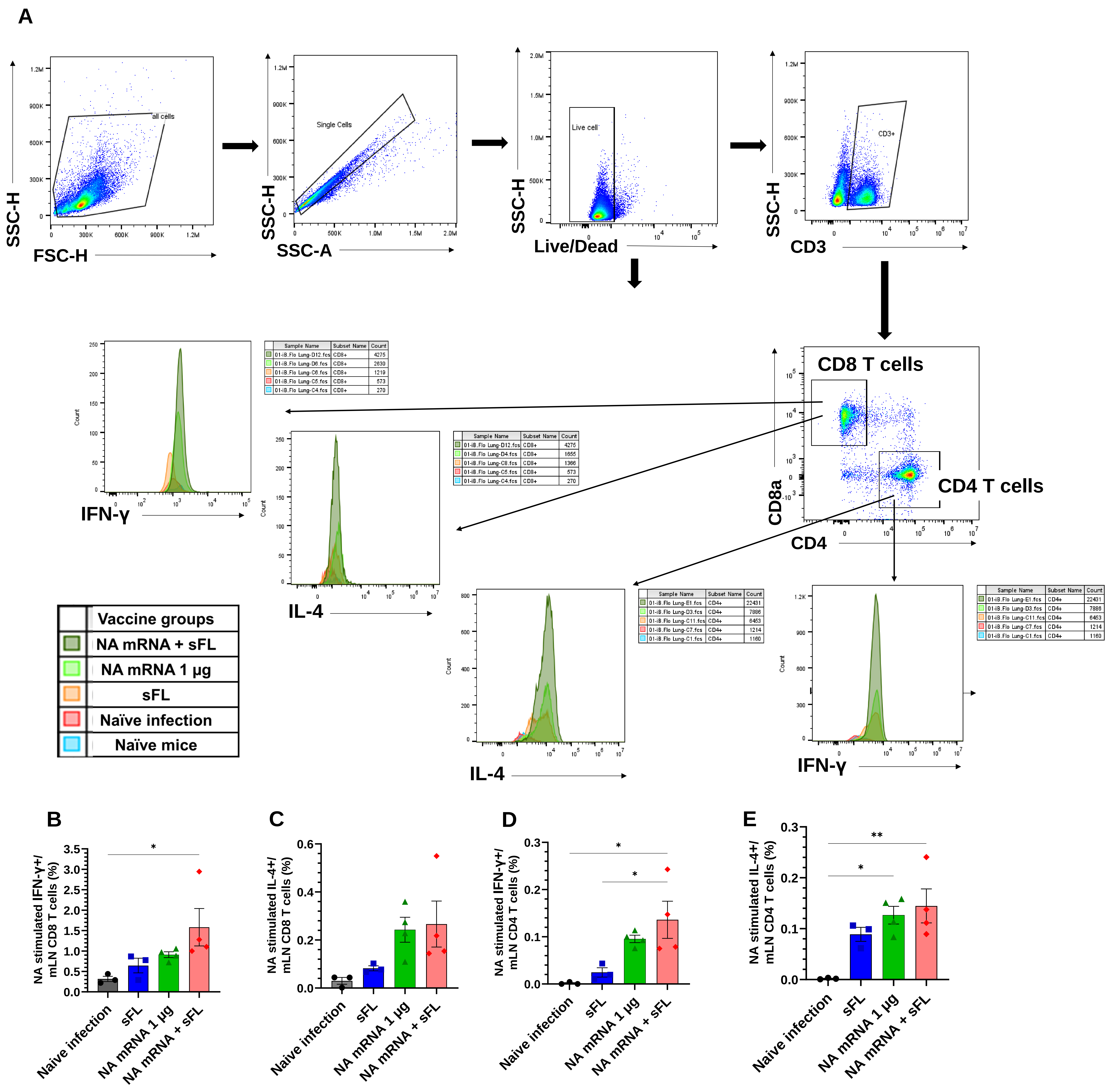

Supplementary Figure S9. Intracellular cytokine staining and flow cytometry grating strategy. IL-4 and IFN-γ produced by CD4 and CD8 T cells gating strategy **(A)**. Frequencies of IL-4 and IFN-γ produced by T cells of mLN upon NA stimulation *in vitro* in lymph nodes (**B-E**). Statistical analysis was performed using one-way ANOVA. P value is significant at $P < 0.05$; $^{*}P < 0.0332$, $^{**}P < 0.0021$, $^{***}P < 0.0002$, $^{****}P < 0.0001$.

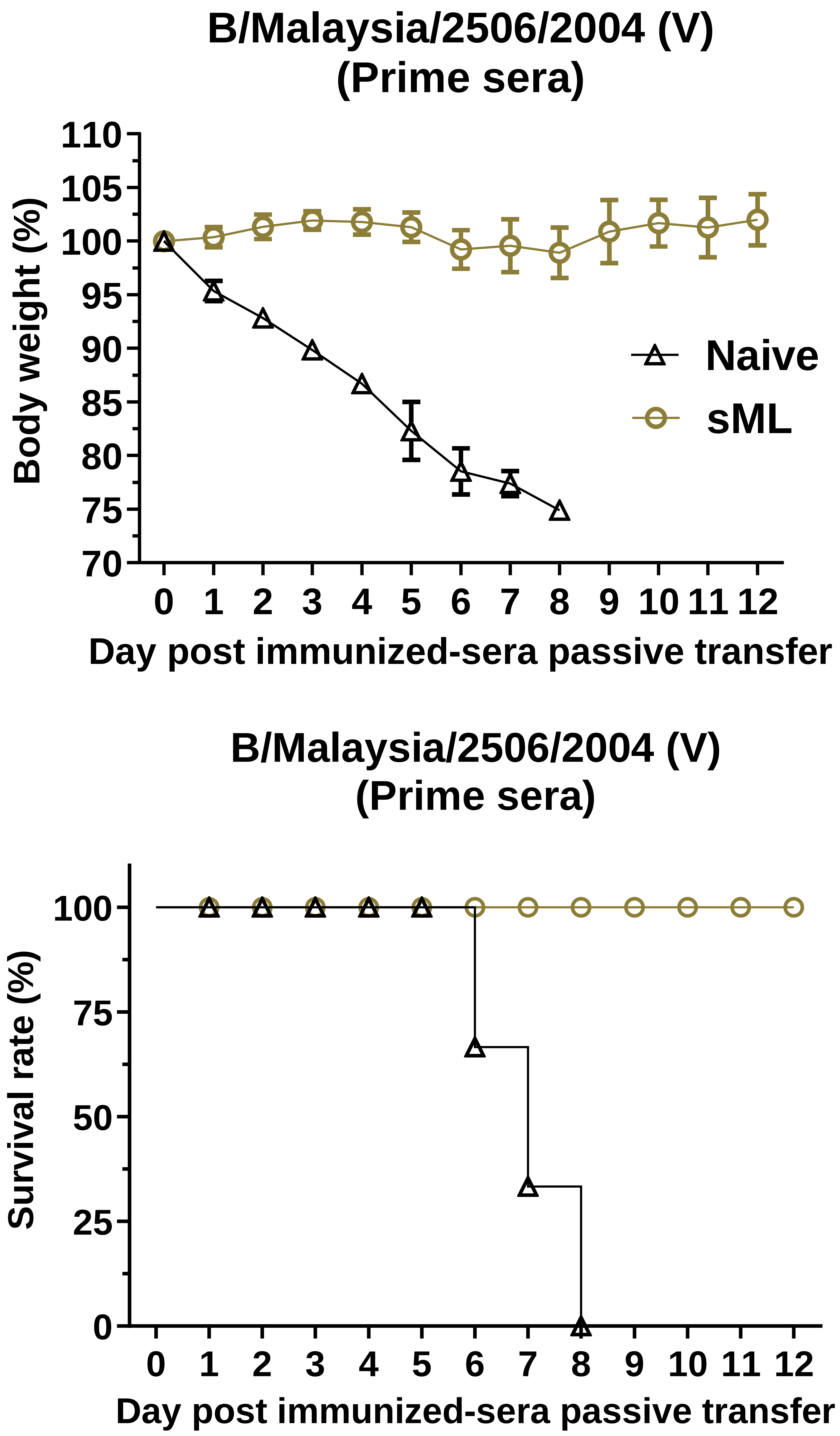

Supplementary Figure S10. Prime antisera of sML vaccination confer protection against homologous virus in naïve mice. Body weight changes and survival rates in naïve mice after (i.n.) inoculation of prime antisera (4X dilution) mixed with B/Malaysia/2004 virus (V) (1.58 x 10^5 EID_{50}). The prime sML (0.15 µg) vaccination is described in Figure 8A.
